# DEAD‐Box Helicase 6 Blockade in Brain‐Derived Aβ Oligomers From Alzheimer's Disease Patients Attenuates Neurotoxicity

**DOI:** 10.1002/mco2.70156

**Published:** 2025-04-24

**Authors:** Xiaoxu Wang, Lu Dai, Na Wu, Donghui Wu, Xinyuan Wang, Xia Meng, Qilei Zhang, Jing Lu, Xiaoxin Yan, Jing Zhang, Baian Chen

**Affiliations:** ^1^ Department of Laboratory Animal Sciences, School of Basic Medical Sciences Capital Medical University Beijing China; ^2^ Laboratory Animal Resource Center Capital Medical University Beijing China; ^3^ Department of Anatomy and Neurobiology Central South University Xiangya School of Medicine Changsha Hunan China; ^4^ Center of Alzheimer's Disease, Beijing Institute of Brain Disorders Capital Medical University Beijing China

**Keywords:** Aβ oligomers, Alzheimer's disease, DDX6, neurotoxicity

## Abstract

There are no effective curative treatments for Alzheimer's disease (AD), the most prevalent form of dementia. Amyloid‐beta (Aβ) oligomers are considered key neurotoxic molecules that trigger AD. Recent studies have shown that direct antibody targeting of Aβ oligomers is beneficial for early AD patients; however, serious side effects (e.g., brain hemorrhage, edema, and shrinkage) persist. Considering that Aβ oligomers readily bind to other proteins, contributing to neurotoxicity and AD onset, those proteins could represent alternative therapeutic targets. However, proteins that bind to Aβ oligomers in the brains of AD patients have not yet been identified. In this study, we identified four proteins (DDX6, DSP, JUP, and HRNR) that bind to Aβ oligomers derived from the brains of AD patients. Intriguingly, among these four proteins, only the blockade of DEAD‐box helicase 6 (DDX6) in human‐derived Aβ oligomers attenuated their neurotoxicity both in vitro and in vivo. Mechanistic analysis revealed that DDX6 promotes the formation of Aβ oligomers, likely due to DDX6 bind to Aβ oligomers at four distinct sites. These findings suggest that DDX6 could serve as a potential therapeutic target to reduce the neurotoxicity of Aβ oligomers in the brain and prevent the progression of AD.

## Graphical abstract

We identified four proteins (DSP, JUP, HRNR, and DDX6) that bind to amyloid‐beta (Aβ) oligomers derived from the brains of Alzheimer's disease (AD) patients. Among these four proteins, only the blockade of DEAD‐box helicase 6 (DDX6) in AD brain‐derived Aβ oligomers attenuated their neurotoxicity. Mechanistic analysis showed that DDX6 promotes the formation of Aβ oligomers, likely due to DDX6 bind to Aβ oligomers at four distinct sites. Thus, DDX6 could serve as a therapeutic target for efforts to reduce the neurotoxicity of Aβ oligomers in the brain and prevent subsequent development of AD.

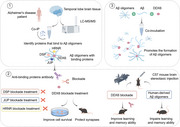

## 1 Introduction

Amyloid‐beta (Aβ) oligomers, soluble polymers formed by the aggregation of Aβ monomers, accumulate in the brains of Alzheimer's disease (AD) patients and contribute to the formation of amyloid plaques [1]. Over the past decade, strong evidence has emerged that Aβ oligomers can cause tau pathology, synaptic dysfunction, neuronal loss, and cognitive impairment [1, 2]. Thus, Aβ oligomers play a central role in AD pathogenesis. Gong et al. reported that Aβ oligomer levels can be up to 70‐fold higher in human AD brains than in healthy human brains [3]. The presence of Aβ oligomers is strongly associated with clinical symptom severity and disease progression [4, 5]. Aβ oligomers induce overexpression of glutamate receptors, leading to tau hyperphosphorylation and oxidative stress [5, 6]. Accordingly, Aβ oligomers are considered a major driver of neuronal degeneration and nerve cell death in AD [7]. Aβ oligomers also trigger neuroinflammation, activating astrocytes, which are key regulators of the neuroinflammatory response. This activation promotes the replication and spreading of toxic Aβ oligomers, causing neuronal damage and exacerbating AD progression [8, 9]. In vitro and in vivo studies have shown that human‐derived Aβ oligomers inhibit hippocampal long‐term potentiation in animal models, impair synaptic plasticity, reduce synapse numbers, and impair learning and memory abilities, ultimately resulting in cognitive dysfunction [10]. Therefore, Aβ oligomers are considered key molecules in AD pathogenesis.

Various Aβ antibody drugs designed to clear Aβ plaques from the brain have shown promising therapeutic effects in animal models (e.g., AD mice). However, Aβ antibody drugs, such as bapineuzumab and solanezumab, have failed to demonstrate efficacy in improving cognitive function during key clinical trials [11, 12, 13]. Although aducanumab received accelerated approval from the United States Food and Drug Administration for the treatment of early‐stage AD, its efficacy in slowing or halting AD progression remains controversial [11, 14]. Therapies targeting Aβ oligomers have shown favorable therapeutic effects in animal experiments. Recent clinical trials have also confirmed that the monoclonal antibody lecanemab, which selectively targets Aβ oligomers, can delay cognitive decline by 27% in early‐stage AD patients [15]. However, some patients have experienced severe side effects, including brain hemorrhage, brain shrinkage, and even death [15, 16]. These effects likely occur because Aβ‐targeting antibody drugs rely on immune cells, such as microglia, to clear Aβ. Excessive or rapid clearance can trigger inflammatory responses, leading to cerebral vascular leakage and rupture [17, 18].

Protein–protein interactions are likely to affect the respective functions of the involved proteins. There is evidence that interactions between Aβ oligomers and other proteins influence the development and progression of AD [19, 20, 21]. Previous targeted studies have shown that interactions involving cellular prion proteins, Aβ oligomers, and Fyn lead to tau hyperphosphorylation and synaptic damage [22]. Interactions between Aβ oligomers and lipid rafts have also been proposed to cause memory loss [23]. The findings thus far indicate that Aβ oligomer neurotoxicity is influenced by the proteins with which these oligomers interact. Therefore, we propose a therapeutic strategy for AD that involves targeting proteins binding to Aβ oligomers, rather than directly targeting the oligomers themselves. However, the proteins that bind to Aβ oligomers in human AD brains remain poorly understood, and the impact of targeting these binding proteins on Aβ oligomer neurotoxicity is unknown.

In support of this approach, as shown in Figure S1, we utilized affinity purification mass spectrometry (AP‐MS) to identify four proteins that bind to human‐derived Aβ oligomers: DEAD‐box helicase 6 (DDX6), desmoplakin (DSP), junction plakoglobin (JUP), and hornerin (HRNR). Through in vitro cellular experiments and in vivo animal studies, we found that among these four binding proteins, only the blockade of DDX6 in human‐derived Aβ oligomers reduced neurotoxicity. Mechanistic studies confirmed a strong binding affinity between DDX6 and Aβ oligomers. Additionally, we discovered that DDX6 promotes the aggregation and formation of Aβ fibrillar plaques and Aβ oligomers through four specific binding sites. Our findings suggest that DDX6 targeting could represent a promising therapeutic approach to prevent Aβ oligomer neurotoxicity during AD development and progression.

## 2 Results

### 2.1 Successful Enrichment and Validation of Aβ Oligomers From AD Patient Brain Tissue Using Co‐Immunoprecipitation

**FIGURE 1:**
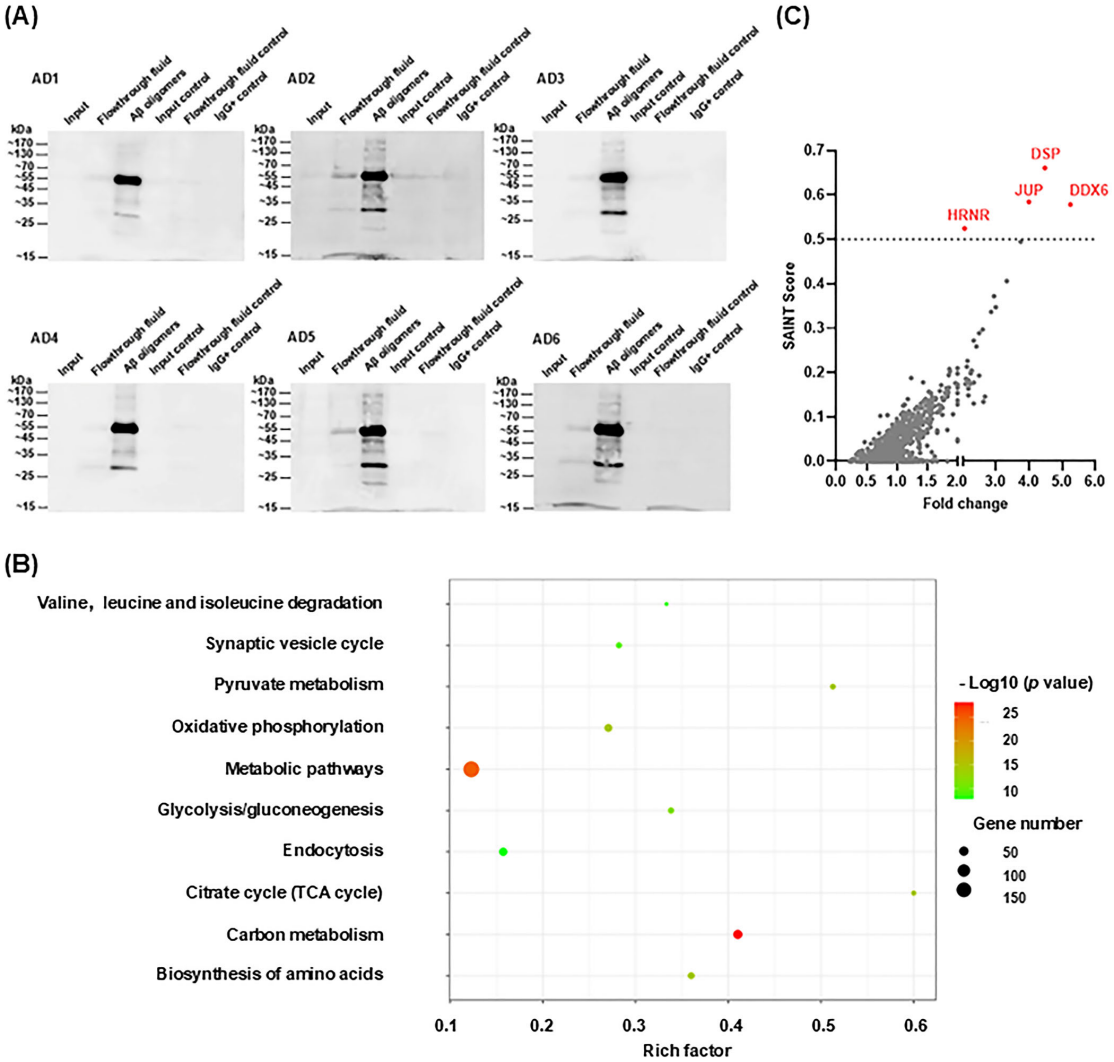
Proteins binding to human‐derived amyloid‐beta (Aβ) oligomers. (A) Western blotting validation of human‐derived Aβ oligomers enriched by co‐immunoprecipitation (co‐IP; AD1–AD6, *n* = 6). Input: total protein; flowthrough fluid: supernatant; Aβ oligomers: Aβ oligomers were enriched and extracted from Alzheimer's disease (AD) patient brain tissue using the A11 antibody; input control: input for the control group; flowthrough fluid control: supernatant for the control group; IgG+ control: IgG. Protein amounts were standardized across all lanes. The third protein lane shows significant enrichment of human‐derived Aβ oligomers. (B) Kyoto Encyclopedia of Genes and Genomes (KEGG) pathway enrichment analysis of all proteins identified by liquid chromatography–tandem mass spectrometry (LC–MS/MS) in human‐derived Aβ oligomers. The *y*‐axis shows pathway names where proteins are enriched. The *x*‐axis represents the rich factor (enrichment factor), calculated as the ratio of annotated proteins in the pathway relative to the background ratio. A higher rich factor indicates greater enrichment. The gene number on the right indicates the number of proteins enriched in each pathway. (C) Analysis of proteins binding to human‐derived Aβ oligomers using SAINTexpress. Each point represents a protein. Proteins with a fold change ≥ 2 and SAINT score > 0.5 are highlighted in red and considered binding partners for human‐derived Aβ oligomers.

In this study, we aimed to enrich and extract Aβ oligomers from the temporal lobe brain tissues of six AD patients (AD1–AD6). Aβ oligomers were enriched and extracted from complex total protein mixtures using co‐immunoprecipitation (co‐IP) with the A11 antibody. The A11 antibody specifically recognizes soluble Aβ42 oligomers but does not react with soluble low‐molecular‐weight Aβ40 or Aβ40 fibrils; it has been confirmed to detect type I Aβ oligomers (dodecamers or larger) with sizes ≥ 50 kDa [24, 25]. Western blotting assays using the A11 antibody confirmed the enrichment of human‐derived Aβ oligomers. As shown in Figure 1A, comparisons were performed using equal amounts of total protein in each lane. Input samples with lower protein concentrations exhibited trace amounts of Aβ oligomers. However, after enrichment via co‐IP, a significant increase in Aβ oligomer content was observed (third protein lane), predominantly at 55 kDa; additional bands appeared at other molecular weights. An isotype control IgG antibody was used to confirm the absence of nonspecific binding. To further characterize human‐derived Aβ oligomers, we used the 6E10 antibody, which recognizes both monomeric and oligomeric forms of Aβ, for detection. The results showed that the corresponding oligomers were identifiable in the co‐IP extract (Figure S2).

### 2.2 Identification of DDX6, DSP, JUP, and HRNR as Novel Binding Partners of Human‐Derived Aβ Oligomers

In this study, we sought to identify proteins that interact with human‐derived Aβ oligomers. Accordingly, we performed liquid chromatography–tandem mass spectrometry (LC–MS/MS, BiotechPack; Shanghai, China) to identify all proteins present in human‐derived Aβ oligomer samples. The LC–MS/MS analysis identified 1070 proteins across 12 co‐IP samples (Table S1 and Figure 1B). As expected, most samples contained tubulin polymerase‐promoting protein (TPPP), which is expressed in hippocampal neurons and fibers in AD patients [26]. TPPP is a known interaction partner for soluble Aβ oligomers [27]; pathogen‐like oligomers produced by TPPP and Aβ oligomers, in conjunction with α‐synuclein, are closely associated with AD pathogenesis and accelerated cognitive dysfunction [28]. Catalase (CAT) and prosaposin (PSAP) were also detected in most co‐IP samples. CAT is a neuroprotective amyloid‐binding protein that directly protects nerves in combination with Aβ [29]. PSAP, a regulator of neuroprotective secretory proteins and lysosomal function, is primarily expressed in Aβ plaques and neurons; it has demonstrated a significant positive correlation with Aβ levels [30]. The detection of TPPP, CAT, and PSAP in the present study supports the reliability of the LC–MS/MS results.

Not all identified proteins bind to Aβ oligomers. Therefore, we used the SAINTexpress statistical method to screen for proteins most likely to interact with human‐derived Aβ oligomers [31, 32, 33]. SAINTexpress assigns probability scores to factors interacting with target proteins, providing a value between 0 and 1, where higher scores indicate a greater likelihood of interaction with Aβ oligomers. Based on the analyzed results, we plotted the fold change on the *x*‐axis and SAINT scores on the *y*‐axis, with each point representing a protein. Proteins meeting the criteria of a SAINT score > 0.5 and fold change ≥ 2 were considered the most likely to bind Aβ oligomers (highlighted in red). Through rigorous analysis, we identified four proteins as binding partners (Figure 1C): DDX6 (SAINT score = 0.5783), DSP (SAINT score = 0.6608), JUP (SAINT score = 0.5846), and HRNR (SAINT score = 0.5246).

### 2.3 Confirmation of Protein‐Aβ Oligomer Interactions Through Co‐IP and Immunohistochemistry Analyses

**FIGURE 2:**
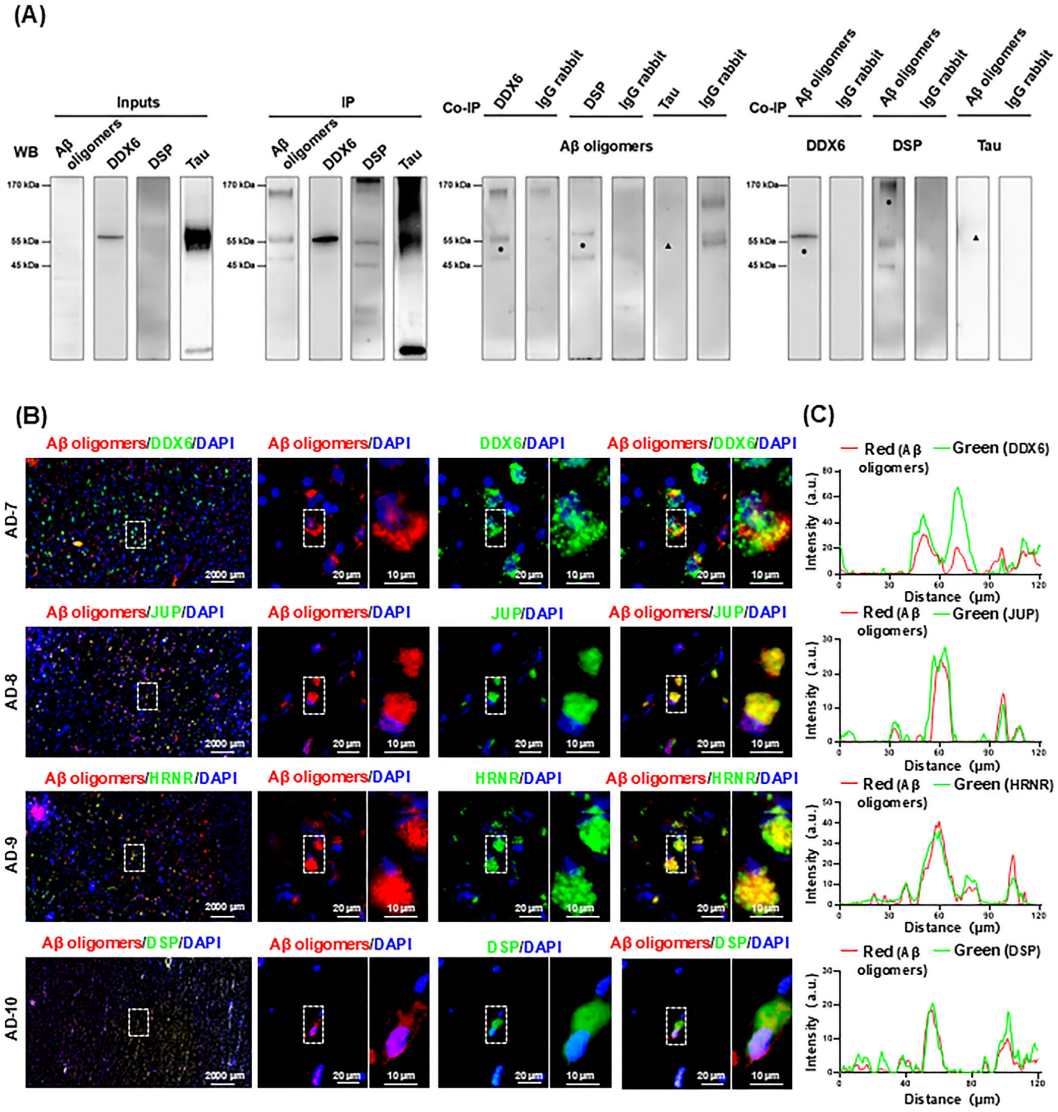
Validation of proteins binding to human‐derived amyloid‐beta (Aβ) oligomers predicted by SAINTexpress analysis via co‐immunoprecipitation (co‐IP) and immunohistochemistry (IHC). (A) Co‐IP showing DEAD‐box helicase 6 (DDX6) and desmoplakin (DSP) binding with Aβ oligomers. Inputs: human‐derived Aβ oligomers, DDX6, DSP, and tau were detected in Alzheimer's disease (AD) brain extracts. Immunoprecipitation (IP): Aβ oligomers, DDX6, DSP, and tau were considerably enriched by using their antibody in AD brain extracts. Co‐IP: Aβ oligomers were detected in the IP extracts of DDX6 and DSP, in addition, DDX6 and DSP were detected in the IP extracts of Aβ oligomers (“
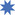
” indicates protein band position). Aβ oligomers were not detected in IP extracts of tau, and tau was not detected in the IP extracts of Aβ oligomers (“▲” indicates the protein band position). IgG rabbit: Isotype control anti‐rabbit IgG antibody was used to eliminate nonspecific binding. (B) The four binding proteins colocalize with Aβ oligomers in AD brain via IHC analysis (AD7–AD10, *n* = 4). Brain tissue sections from AD patients were stained for Aβ oligomers (red); DDX6, junction plakoglobin (JUP), hornerin (HRNR), and DSP (green); and 4',6‐diamidino‐2‐phenylindole (DAPI (blue). (C) Graphs on the right show the corresponding intensity scans of Aβ oligomers and the four proteins over a 120‐µm line extending from the left to the right side of stained brain tissue.

After rigorous data analysis with SAINTexpress, four proteins (DDX6, DSP, JUP, and HRNR) were identified as potential Aβ oligomer‐binding partners. To validate these findings, we selected DSP and DDX6 as positive controls for co‐IP analysis; tau proteins were used as negative controls. Input samples, IP, co‐IP extracts, and IgG controls were adjusted to ensure equal amounts of protein for comparison. We found that Aβ oligomers, DDX6, DSP, and tau proteins were all present in the brain tissues (i.e., input samples) of AD patients (Figure 2A). The IP enrichment effect was substantial for Aβ oligomers, DDX6, DSP, and tau proteins. In the co‐IP extracts for DDX6 and DSP, Aβ oligomers were detected at approximately 55 and 30 kDa, respectively (indicated by “
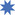
”). DDX6 and DSP were also detected in Aβ oligomer co‐IP extracts at approximately 54 and 220 kDa, respectively (indicated by “
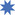
”). No apparent binding was observed between tau protein and Aβ oligomers (indicated by “▲”). Isotype control IgG was used to exclude nonspecific binding (Figure 2A). Detailed protein band results for the entire polyvinylidene fluoride membrane are provided in Figures S3 and S4. These results collectively demonstrate the binding of DDX6 and DSP to Aβ oligomers.

To further validate the binding interactions between Aβ oligomers and the four proteins, we performed immunohistochemistry (IHC) on brain sections from four AD patients (AD7–AD10). We analyzed the colocalization of Aβ oligomers (red) with DDX6, JUP, HRNR, and DSP (all green), as well as 4',6‐diamidino‐2‐phenylindole (DAPI)‐stained nuclei (blue). Costaining revealed strong colocalization of Aβ oligomers with DDX6, JUP, HRNR, and DSP (Figure 2B). We also performed fluorescence intensity scanning from left to right across stained brain tissue slices (120 µm). The results showed consistent overlap in fluorescence intensity between Aβ oligomers and the four binding proteins (Figure 2C). The combined results from co‐IP and IHC confirm that these four proteins can bind to Aβ oligomers.

### 2.4 DDX6 Blockade Attenuates Human‐Derived Aβ Oligomer‐Induced Neuronal Cell Death and Toxicity In Vitro

**FIGURE 3:**
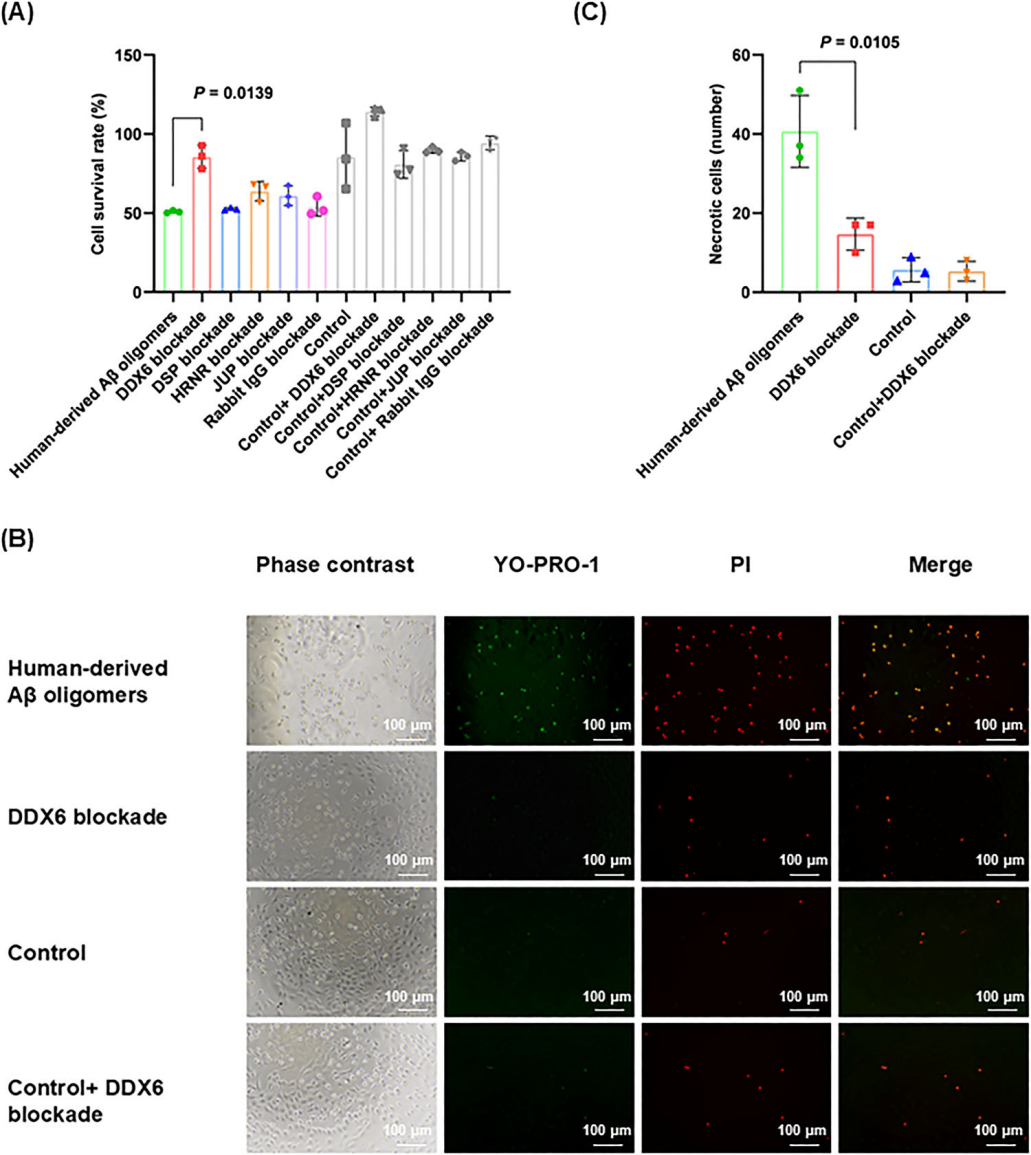
DEAD‐box helicase 6 (DDX6) blockade in human‐derived amyloid‐beta (Aβ) oligomer extracts attenuates neurotoxicity in vitro. (A) Cell counting kit‐8 (CCK‐8) cytotoxicity assay (12 groups; *n* = 3). Cell survival rate (%) = (experimental mean absorbance − blank mean absorbance)/(control mean absorbance − blank mean absorbance) × 100%. (B) YO‐PRO‐1/PI double staining (four groups; *n* = 3). YO‐PRO‐1 (green) indicates apoptosis; YO‐PRO‐1 (green) + propidium iodide (PI) (red) indicates necrosis. (C) *Y*‐axis: number of necrotic cells. Statistical analyses were performed using unpaired Student's *t*‐tests with a significance threshold of *p* < 0.05. Data are expressed as the mean ± standard deviation (SD).

To investigate whether the four identified binding proteins (DDX6, DSP, JUP, and HRNR) influence Aβ oligomer neurotoxicity, we performed cell‐level validation by blocking their expression in human‐derived Aβ oligomer extracts (2 µg). Four experimental treatments were established: (1) human‐derived Aβ oligomers; (2) human‐derived Aβ oligomer + anti‐binding protein/IgG antibody (DDX6 blockade, DSP blockade, HRNR blockade, JUP blockade, or rabbit IgG blockade); (3) solvent control (control); (4) solvent control + anti‐binding protein/IgG antibody (control + DDX6 blockade, control + DSP blockade, control + JUP blockade, control + HRNR blockade, or control + rabbit IgG blockade). These treatments were applied to the HT‐22 mouse hippocampal neuronal cell line for 24 h. After treatment, cells were incubated with the cell counting kit‐8 (CCK‐8) reagent for 3 h. Enzymatic labeling was used to determine the optical density at 450 nm. As shown in Figure 3A, among the four binding proteins, only the blockade of DDX6 in human‐derived Aβ oligomer extracts significantly reduced the toxic effects of these extracts on nerve cells (*p* = 0.0139). Neither the solvent control nor the antibody control treatments had significant effect on the cells.

To further investigate the specific mechanisms by which DDX6 affects the neuronal toxicity of Aβ oligomers, we examined apoptosis and necrosis using the YO‐PRO‐1 (green fluorescence)/PI (red fluorescence) detection kit. This kit measured the toxic effects of human‐derived Aβ oligomers and DDX6 blockade on HT‐22 cells. Apoptotic cells displayed green fluorescence; strong overlap of the two fluorescence signals indicated necrosis, rather than apoptosis. The concentrations used for each group were consistent with those in the CCK‐8 cytotoxicity experiment. We found that the blockade of DDX6 in human‐derived Aβ oligomer extracts resulted in a reduced number of necrotic cells (*p* = 0.0105; Figure 3B,C). Thus, our in vitro assays demonstrated that DDX6 blockade in human‐derived Aβ oligomer extracts attenuated Aβ oligomer neurotoxicity.

### 2.5 DDX6 Blockade Protects Against Human‐Derived Aβ Oligomer‐Induced Axonal, Synaptic, and Dendritic Damage in Mouse Brains

**FIGURE 4:**
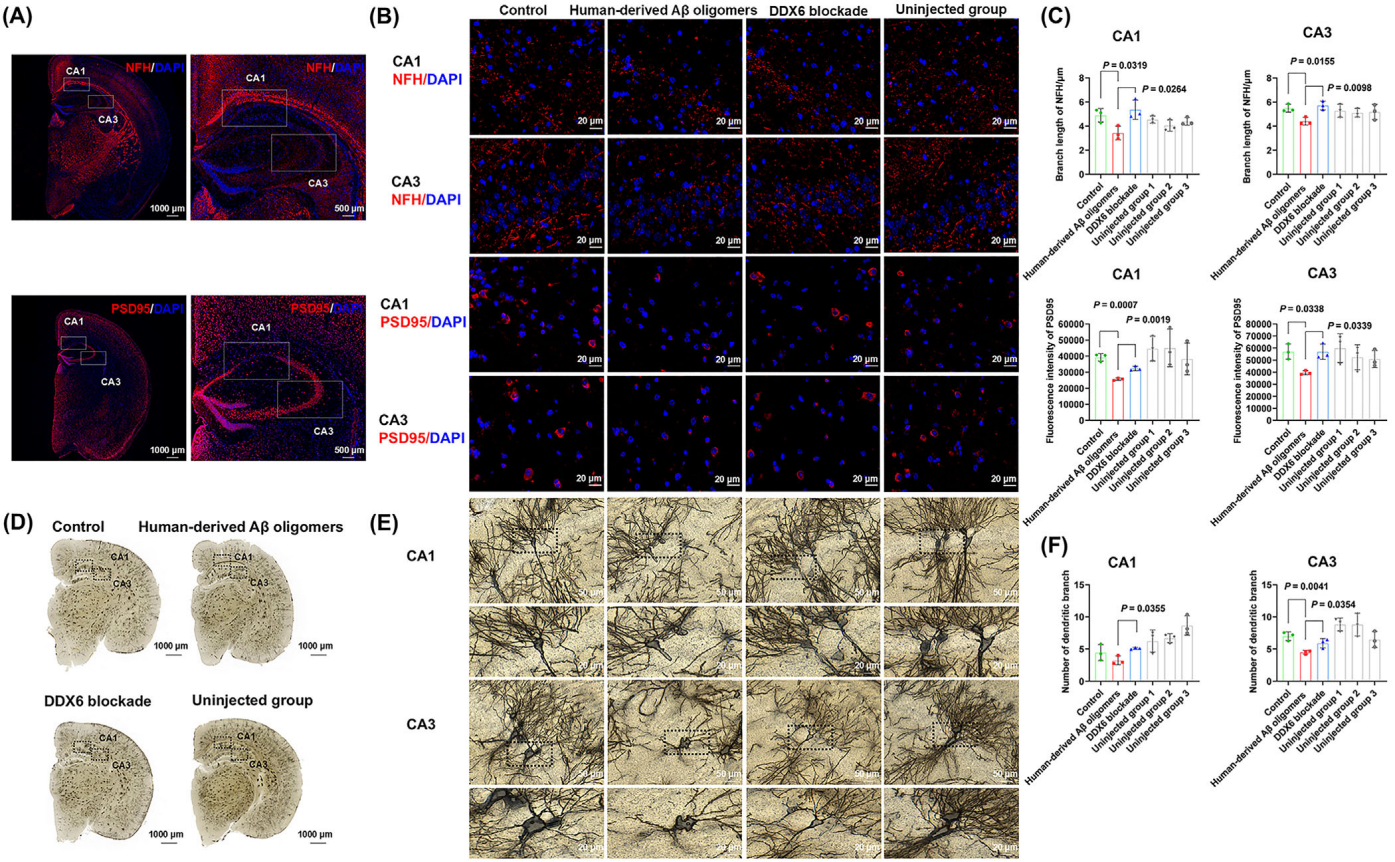
DEAD‐box helicase 6 (DDX6) blockade in human‐derived amyloid‐beta (Aβ) oligomers attenuates neurotoxicity in vivo (three groups; *n* = 6). (A–C) DDX6 blockade in human‐derived Aβ oligomers reduces axonal and synaptic loss. Neurofilament heavy (NFH)/postsynaptic density protein‐95 (PSD95; red) and 4',6‐diamidino‐2‐phenylindole (DAPI; blue)‐stained confocal images of the CA1 and CA3 regions in mouse brains (*n* = 3). White‐dotted squares indicate the CA1 and CA3 regions of the hippocampus. (C) *Y*‐axis: mean NFH (axon) branch length and fluorescence intensity of PSD95 (synapses). “Uninjected group 1, 2, and 3” represent homologous control results from the uninjected right side of the same mouse brain. (D, E) Golgi staining (*n* = 3). DDX6 blockade in human‐derived Aβ oligomers reduces dendritic loss. Morphology of neuronal dendrites in the CA1 and CA3 regions (20×, 40×). (F) *Y*‐axis: number of dendritic branches. Statistical analyses were performed using unpaired Student's *t*‐tests with a significance threshold of *p* < 0.05. Data are expressed as the mean ± standard deviation (SD).

We used a mouse model to further explore whether DDX6 blockade could attenuate the neurotoxicity of human‐derived Aβ oligomers. Eighteen C57BL/six mice were divided into three groups for brain stereotaxic injection: control, human‐derived Aβ oligomers, and DDX6 blockade (0.5 µg, *n* = 6 per group). After 1 month, mouse brains were collected for analysis by IHC (Figure 4A,B) and Golgi staining (Figure 4D,E). Statistical analysis (Figure 4C) revealed statistically significant differences in mean axon (neurofilament heavy [NFH]) branch lengths between the control and human‐derived Aβ oligomer groups (*p* = 0.0319 in cornuammonis [CA] 1 and *p* = 0.0155 in CA3). Furthermore, mean NFH branch lengths significantly differed between the human‐derived Aβ oligomer and DDX6 blockade groups (*p* = 0.0264 in CA1 and *p* = 0.0098 in CA3). We also observed significant differences in the mean fluorescence intensity of synapses (postsynaptic density protein‐95 [PSD95]) between the control and human‐derived Aβ oligomer groups (*p* = 0.0007 in CA1 and *p* = 0.0338 in CA3), as well as between the human‐derived Aβ oligomer and DDX6 blockade groups (*p* = 0.0019 in CA1 and *p* = 0.0339 in CA3). Golgi staining (Figure 4F) showed a significant difference in the number of dendritic branches between the human‐derived Aβ oligomer and control groups (*p* = 0.0041 in CA3). The number of dendritic branches also significantly differed between the human‐derived Aβ oligomer and DDX6 blockade groups (*p* = 0.0355 in CA1 and *p* = 0.0354 in CA3). No statistically significant differences were observed between the uninjected groups (the right brains of the three mouse groups, serving as nonsurgical controls) and the control group, indicating that the injection procedure did not significantly affect these parameters. Overall, these results suggest that DDX6 blockade in human‐derived Aβ oligomers can mitigate axonal, synaptic, and dendritic loss.

### 2.6 DDX6 Blockade Alleviates Cognitive Deficits in Human‐Derived Aβ Oligomer‐Treated Mice Across Multiple Behavioral Tests

**FIGURE 5:**
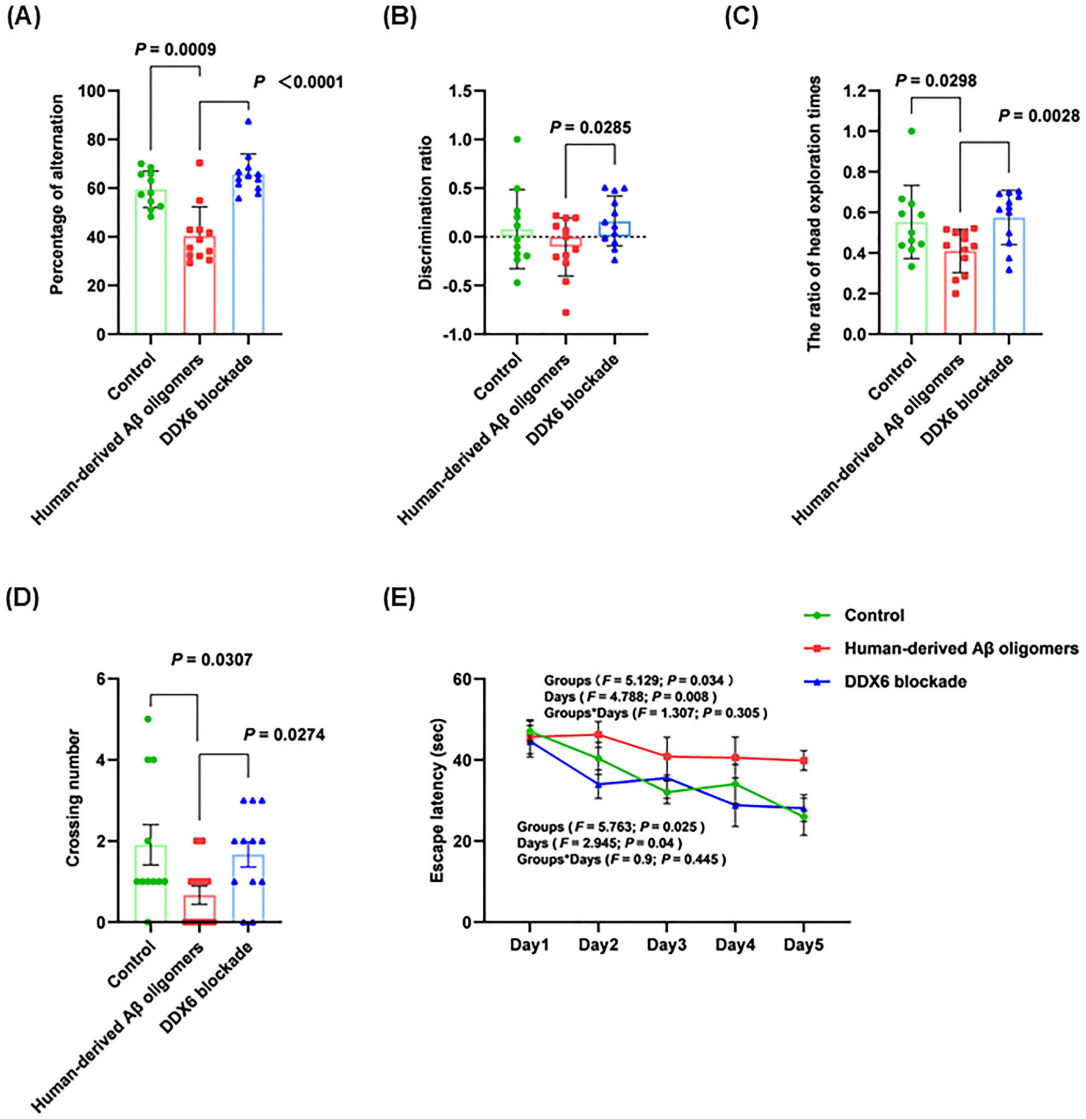
DEAD‐box helicase 6 (DDX6) blockade in human‐derived amyloid‐beta (Aβ) oligomers improves learning and memory abilities in mice. Cognitive behavioral tests (Control: *n* = 11; human‐derived Aβ oligomers: *n* = 12; DDX6 blockade: *n* = 12). (A) Y‐maze test. DDX6 blockade improved learning and working memory abilities in mice. Percentage of alternation = total alternations/(total number of entries – 2) × 100%. (B, C) Novel object recognition test. Learning and short‐term memory abilities in mice were significantly improved after DDX6 blockade. Discrimination ratio (DR) = (time exploring novel object − time exploring familiar object)/(time exploring novel object + time exploring familiar object). Ratio of head exploration times = number of head explorations of the novel object/(number of head explorations of the novel object + number of head explorations of the familiar object). (D) Morris water maze. Platform crossings for mice in the human‐derived Aβ oligomer group were statistically lower than those in the DDX6 blockade group. Statistical analyses were performed using unpaired Student's *t*‐tests with a significance threshold of *p* < 0.05. Data are expressed as the mean ± standard deviation (SD). (E) Changes in escape latency of mice in different groups (Days 1–5). The escape latency of the human‐derived Aβ oligomer group remained high on each testing day, while the escape latency of the other two groups gradually decreased. Statistical analyses were performed using two‐factor repeated measures analysis of variance.

To determine whether DDX6 blockade in human‐derived Aβ oligomers affects learning and memory abilities, we performed brain stereotactic injections in three groups of C57BL/６ mice: control (*n* = 11), human‐derived Aβ oligomers (*n* = 12), and DDX6 blockade (*n* = 12). Learning and memory abilities were assessed on Days 7, 14, and 21 postinjection using the Y‐maze, novel object recognition, and Morris water maze tests. In the Y‐maze test (Figure 5A), the human‐derived Aβ oligomer group showed a significant decrease in the percentage of alternation (*p* = 0.0009). In contrast, the DDX6 blockade group exhibited a significant increase in the percentage of alternation (*p* < 0.0001), indicating improved learning and working memory abilities after DDX6 blockade. The novel object recognition test (Figure 5B,C) showed that the discrimination ratio (DR) and the ratio of head exploration times were significantly higher in the DDX6 blockade group than in the human‐derived Aβ oligomer group (*p* = 0.0285, *p* = 0.0028). These results indicate significant improvements in learning and short‐term memory abilities in the DDX6 blockade group. Importantly, the Morris water maze test (Figure 5D) revealed a significant decrease in the number of platform crossings in the human‐derived Aβ oligomer group (*p* = 0.0307). Conversely, the DDX6 blockade group exhibited a significant increase in the number of platform crossings (*p* = 0.0274). Figure 5E shows the escape latency trends for the three mouse groups over 5 days of training. The escape latency of the human‐derived Aβ oligomer group remained high on each testing day, whereas escape latency in the DDX6 blockade and control groups gradually decreased. Escape latency was significantly longer in the human‐derived Aβ oligomer group than in the control group (groups: *F* = 5.129, *p* = 0.034; days: *F* = 4.788, *p* = 0.008; groups × days: *F* = 1.307, *p* = 0.305; Table S2). Escape latency was significantly shorter in the DDX6 blockade group than in the human‐derived Aβ oligomer group (groups: *F* = 5.763, *p* = 0.025; days: *F* = 2.945, *p* = 0.04; groups × days: *F* = 0.9, *p* = 0.445; Table S2). These results clearly indicate that DDX6 blockade in human‐derived Aβ oligomers improves learning and memory abilities in mice.

### 2.7 Multiple Binding Assays Demonstrate Specific, High‐Affinity Binding Between DDX6 and Aβ Oligomers

**FIGURE 6:**
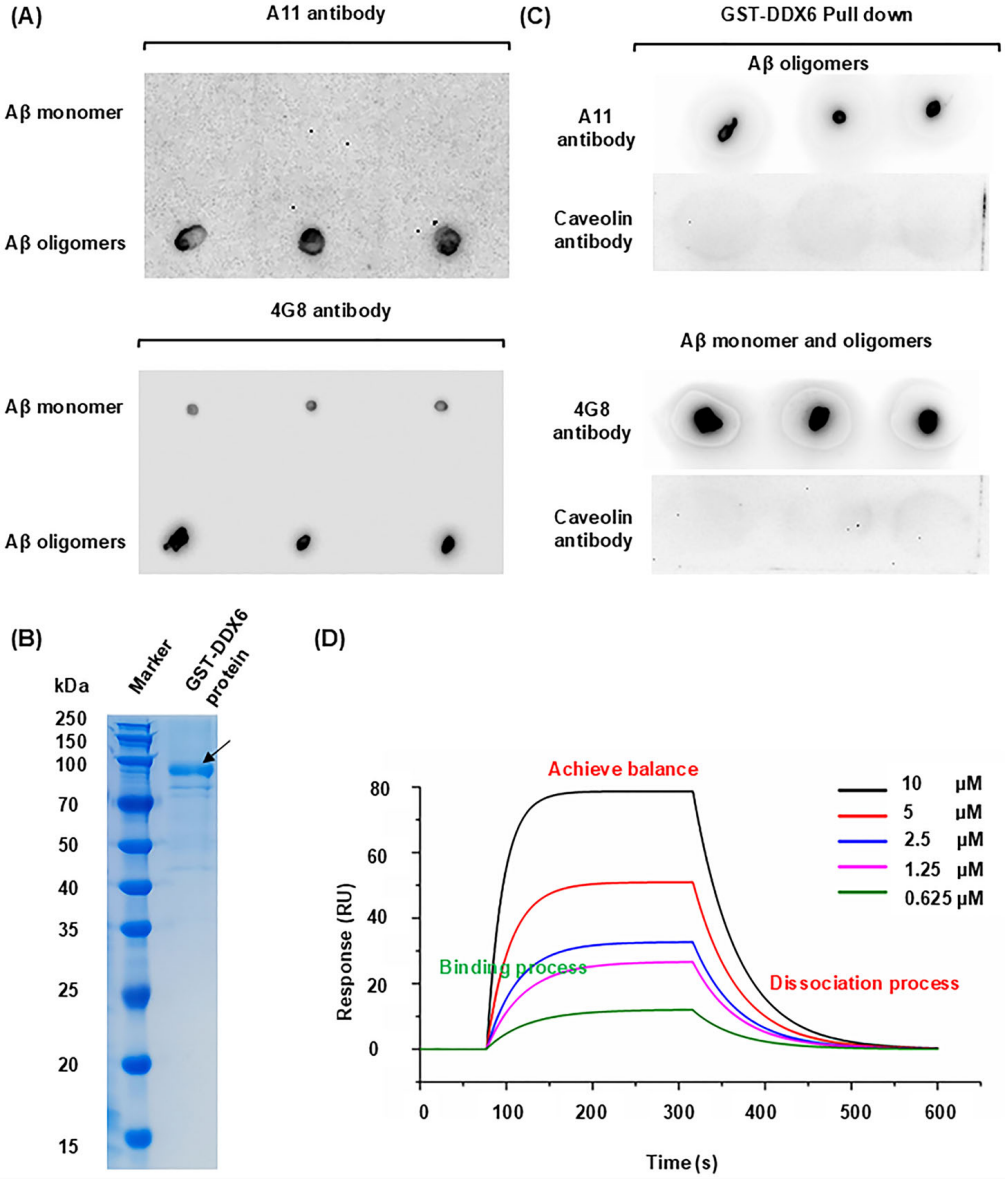
DEAD‐box helicase 6 (DDX6) and amyloid‐beta (Aβ) oligomers show strong affinity. (A) Dot blotting (*n* = 3). The A11 antibody (recognizing Aβ oligomers) and the 4G8 antibody (recognizing both Aβ monomers and Aβ oligomers) confirmed the successful production of Aβ42 oligomers. (B) GST‐DDX6 protein assessed by sodium dodecyl sulfate–polyacrylamide gel electrophoresis. (C) GST pulldown (*n* = 3). GST‐DDX6 successfully pulled down Aβ42 oligomers, whereas caveolin (control) was not detected. (D) Surface plasmon resonance (SPR) assay. DDX6 and Aβ42 oligomers exhibit specific binding in vitro. *X*‐axis: time (s), *Y*‐axis: resonance units (RU). The OpenSPR sensorgram shows the binding, equilibration, and dissociation of DDX6 with Aβ42 oligomers at different concentrations. RU values were proportional to the concentration of DDX6 on the chip surface.

We conducted a series of in vitro experiments to investigate the mechanism by which DDX6 blockade affects Aβ oligomer neurotoxicity. First, to determine whether recombinant DDX6 exhibits strong binding affinity for Aβ42 oligomers, we performed pulldown and surface plasmon resonance (SPR) assays. Dot blotting with anti‐oligomer A11 and 4G8 antibodies, which recognize Aβ oligomers and monomers, respectively, confirmed the successful preparation of Aβ42 oligomers (Figure 6A). We obtained 0.2 mg of recombinant glutathione‐S‐transferase (GST)‐DDX6 peptide at a concentration of 0.18 mg/mL (Figure 6B). Using GST‐labeled DDX6 protein, we successfully pulled down Aβ42 oligomers (Figure 6C); caveolin protein (a negative control) did not bind to DDX6. These results demonstrate that DDX6 exhibits specific binding to Aβ42 oligomers in vitro. Next, we used SPR to assess the affinity between DDX6 and Aβ42 oligomers. DDX6 binding, equilibration, and dissociation were measured using various concentrations of Aβ42 oligomers (10 µM, 5 µM, 2.5 µM, 1.25 µM, and 0.625 µM). The kinetic data generally showed good resonance unit (RU) values, which were directly proportional to the DDX6 concentration on the chip surface (Figure 6D). By calculating the binding constant (Ka, 1/(*M* × *s*) = 3.42 × 10^−3^) and dissociation constant (Kd, (1/*s*) = 1.94 × 10^−2^), we determined that DDX6 and Aβ42 oligomers had a high binding affinity constant (KD = 5.67 µM; Table S3). Therefore, our pulldown and SPR results demonstrated that DDX6 binds to Aβ oligomers with strong affinity.

### 2.8 Mechanistic Analysis Reveals That DDX6 Promotes Aβ Aggregation Through Four Specific Binding Sites

**FIGURE 7:**
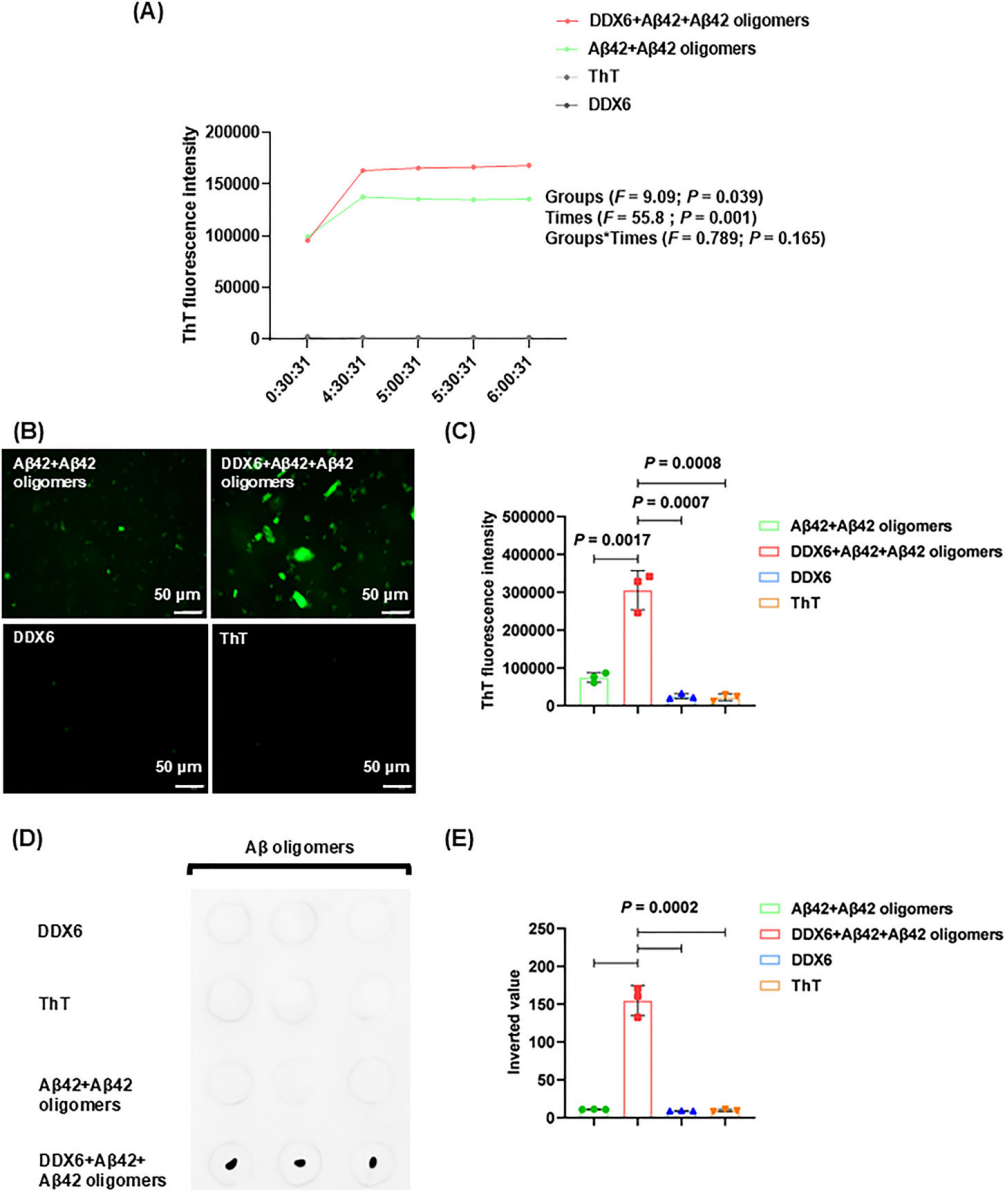
DEAD‐box helicase 6 (DDX6) promotes the formation and aggregation of amyloid‐beta (Aβ) fibrillar plaques and Aβ oligomers. A. Real‐time quaking‐induced conversion (RT‐QuIC) assay (four groups, *n* = 3). Thioflavin T (ThT) fluorescence intensity in the DDX6 + Aβ42 + Aβ42 oligomer group was higher than in the Aβ42 + Aβ42 oligomer group. The *X*‐axis represents time, with shaking every 30 min, and the *Y*‐axis represents ThT fluorescence intensity measured at excitation and emission wavelengths of 440 and 480 nm, respectively. Statistical analyses were performed using two‐way repeated measures analysis of variance. (B, C) ThT‐binding assay (*n* = 3). Inverted microscopy was used to visualize fibrillar plaque content and ThT fluorescence intensity in the four groups. (D, E) Dot blotting was used to detect Aβ oligomers content in the four groups (*n* = 3). Statistical analyses were performed using unpaired Student's *t*‐tests with a significance threshold of *p* < 0.05. Data are expressed as the mean ± standard deviation (SD).

**FIGURE 8:**
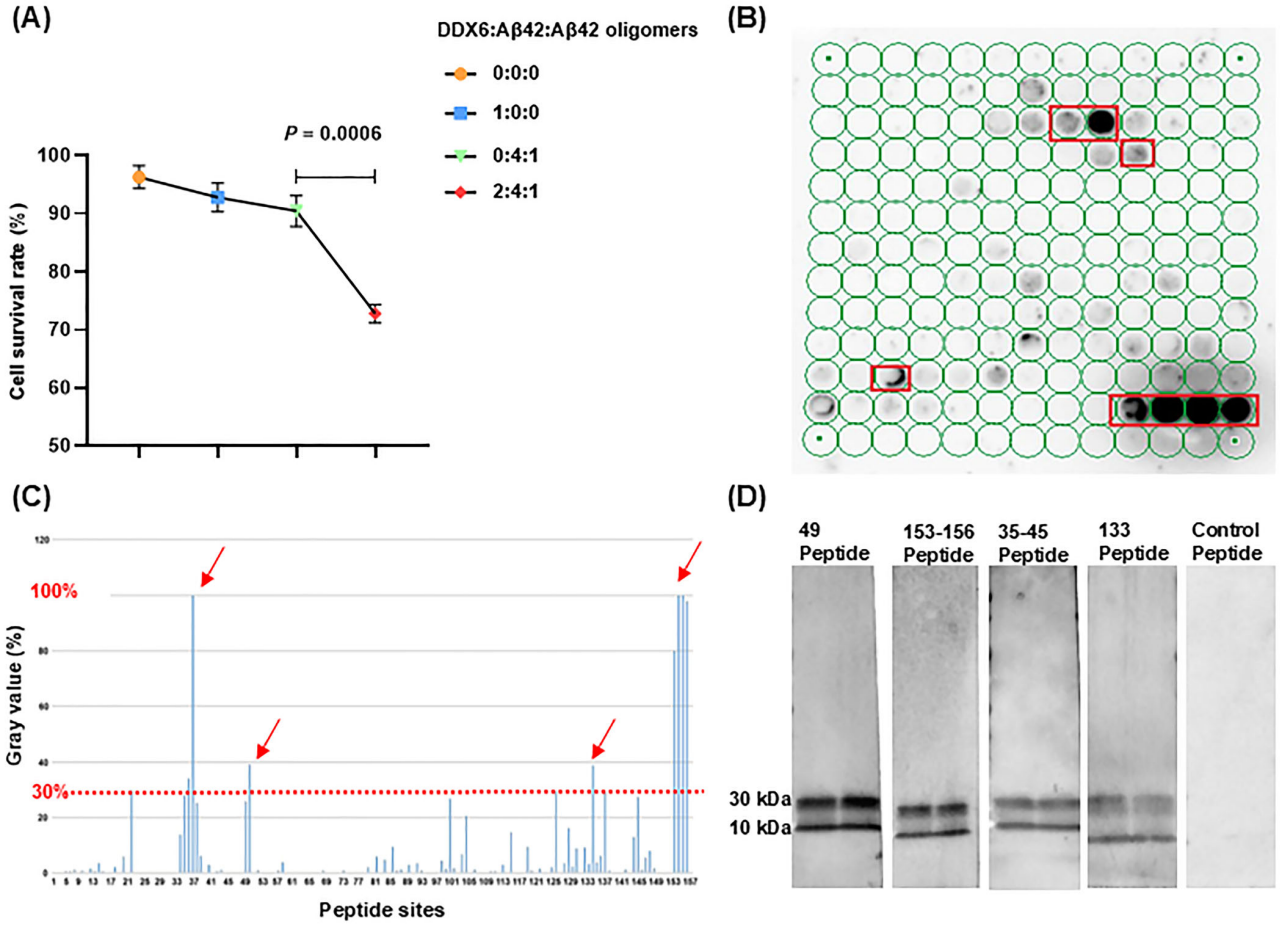
DEAD‐box helicase 6 (DDX6) exacerbates amyloid‐beta (Aβ) oligomer neurotoxicity. (A) Cell counting kit‐8 (CCK‐8) cytotoxicity assay (*n* = 3). DDX6, Aβ42, and Aβ42 oligomers were combined at different concentrations (0:0:0, 1:0:0, 0:4:1, and 2:4:1). (B) Binding sites between DDX6 and Aβ42 oligomers are indicated by red borders. (C) DDX6 promotes the formation of Aβ oligomers in a site‐specific manner. Total Lab 100 analysis software was used to calculate the percentage of gray values for 157 polypeptide spots. Red arrows indicate binding peptide segments with gray values > 30%. (D) Biotin‐labeled short peptides corresponding to the four binding sites demonstrated binding to Aβ42 oligomers.

We characterized the molecular mechanisms by which DDX6 influences Aβ oligomer neurotoxicity using real‐time quaking‐induced conversion (RT‐QuIC) to determine whether DDX6 promotes the formation of Aβ fibrillar plaques and oligomers. The experiment consisted of four treatment groups: (1) DDX6 (10 µM) + Aβ42 (20 µM) + Aβ42 oligomers (5 µM), (2) Aβ42 (20 µM) + Aβ42 oligomers (5 µM), (3) thioflavin T (ThT; 20 µM), and (4) DDX6 (10 µM). We found that the addition of DDX6 to the Aβ42 and Aβ42 oligomer mixture increased green fluorescence intensity at five time points (Figure 7A and Table S4). ThT fluorescence intensity was higher in the DDX6 + Aβ42 + Aβ42 oligomers group than in the Aβ42 + Aβ42 oligomers group (groups: *F* = 9.09, *p* = 0.039; times: *F* = 55.8, *p* = 0.001; groups × times: *F* = 0.789, *p* = 0.428). Fluorescence microscopy (Figure 7B,C) revealed significantly stronger fluorescence intensity in the DDX6 + Aβ42 + Aβ42 oligomers group (*p* = 0.0017), whereas control groups containing only DDX6 or ThT showed no effects on fluorescence intensity (*p* = 0.0007, *p* = 0.0008). These results suggest that DDX6 promotes the formation of Aβ fibrillar plaques. Next, we performed dot blotting assays (Figure 7D,E) on the four treatment groups using the A11 antibody. The results showed significantly higher levels of Aβ42 oligomers in the DDX6 + Aβ42 + Aβ42 oligomers group compared with the Aβ42 + Aβ42 oligomers group (*p* = 0.0002) and the control groups (*p* = 0.0002). These findings indicate that DDX6 promotes the formation of Aβ42 oligomers. We also assessed the cytotoxic effects of the four treatment groups on HT‐22 cell cultures using CCK‐8 assays. The addition of the DDX6 + Aβ42 + Aβ42 oligomers group resulted in a significant decrease in cell survival (*p* = 0.0006), indicating that DDX6 promotes the formation of Aβ oligomers and exacerbates their neurotoxicity (Figure 8A). Subsequently, we investigated how DDX6 promotes Aβ oligomer formation using peptide chip microarray technology to identify binding sites between DDX6 and Aβ42 oligomers. Peptide 1–157 was generated from the DDX6 protein sequence (UniProt P26196: human DDX6). Color spots highlighted within a red frame represent meaningful binding sites (Figure 8B). Specific gray values were calculated using Total Lab 100 (Nonlinear Dynamics, Newcastle upon Tyne, UK). The results showed robust color spots on peptide array chips with values exceeding 30%, including sites 34, 35, 49, 133, and 153–156 (Figure 8C). Previous studies have suggested that values > 30% indicate effective binding sites [34, 35]. The four specific binding sites corresponded to the following peptide sequences: (1) 34–45: DYCLKRELLMGIFEMGWE, (2) 49: GKSGAYLIPLLERLD, (3) 133: GIDIQAVNVVINFDF, and (4) 153–156: IKPIPSNIDKSLYVAEYHSEPVED. To confirm the accuracy of the peptide array chip results, we synthesized four biotin‐labeled peptide probes corresponding to these specific sites, along with a control peptide (KPYEINLMEELTLKG). These peptides were separately incubated with Aβ42 oligomers. We found that all four binding peptides interacted with Aβ oligomers, as indicated by protein bands at molecular weights of 30 and 10 kDa (Figure 8D). In contrast, the control peptide did not interact with Aβ oligomers. Therefore, our RT‐QuIC, dot blotting, and peptide array chip analyses demonstrated that DDX6 promotes the formation of Aβ fibrillar plaques and Aβ oligomers, likely due to DDX6 bind to Aβ oligomers at four distinct sites.

## 3 Discussion

In this study, we employed an AP‐MS proteomics approach to identify proteins that bind to human‐derived Aβ oligomers. To our knowledge, this study represents the first comprehensive analysis of binding partners for human‐derived Aβ oligomers. The results provide valuable biomarker candidates for future investigations into novel disease mechanisms and therapies for AD. Although not all identified proteins may genuinely interact with Aβ oligomers, our rigorous data analysis confirmed four proteins—DSP, JUP, DDX6, and HRNR—as exhibiting strong binding to human‐derived Aβ oligomers. Importantly, these proteins have not previously been associated with Aβ oligomers. DSP, a molecule involved in cell adhesion, serves as a functional bridge‐specific component with intermediate filaments. It is a key downstream molecule of Bcl11b during hippocampal development. Bcl11b induces postmitotic neuronal mutations that prevent integration within the hippocampus, leading to impaired spatial learning and memory. Restoration of DSP expression can reverse this damage [36]. JUP, also known as γ‐catenin, is a cytoplasmic component of adherens junctions and desmosomes [37]. Intriguingly, there is evidence of increased JUP expression among patients with semantic dementia, particularly in the hippocampus. HRNR is reportedly upregulated in eosinophilic neuronal inclusions [38], but its role in AD has not been extensively studied. DDX6, also known as PCK or P54, is a core component of membraneless cytoplasmic substructures where RNA and proteins undergo functional regulation [39]. DDX6 functions as a translational repressor, modulating mRNA translation in yeast, as well as in the hippocampal neurons of humans and mice [40].

When examining the effects of these four proteins on human‐derived Aβ oligomer neurotoxicity, we found that DDX6 blockade attenuated neurotoxicity; this finding was confirmed in both cellular and animal models. Therefore, DDX6 has the potential to serve as a novel therapeutic target for preventing AD onset. We conducted in vitro experiments using pulldown and SPR techniques to confirm the specific binding between recombinant DDX6 and Aβ oligomers. ThT‐binding assays and dot blotting further demonstrated that DDX6 promotes the formation of Aβ oligomers and binds to these oligomers at four specific sites. We speculate that DDX6 binding to Aβ oligomers alters their status, inducing further oligomer formation by recruiting Aβ monomers. The results of previous studies suggest that amyloid fiber proliferation occurs through a two‐step nucleation mechanism involving oligomer production. These oligomers transition into fibrous structures and eventually dissociate into monomers. Oligomers undergo repeated formation–dissociation cycles before ultimately transforming into species capable of growing into new fibrils [41]. Based on these findings, we propose that DDX6 promotes the initial or early‐stage formation and aggregation of Aβ oligomers, thereby accelerating AD progression.

In recent years, scientific research concerning protein–RNA phase separation has received substantial attention. A team of researchers from the Institute of Biochemistry at the Federal Institute of Technology in Zurich, Switzerland, revealed that DDX6 is a highly ATP‐dependent enzyme that regulates the fusion and release of RNA by utilizing energy provided by ATP. This activity directs RNA through the phase separation chamber and promotes the comprehensive regulation of RNA–protein complex phase separation [42]. Reduced expression of purine‐binding protein A (PURA) leads to decreased DDX6 expression, which subsequently affects RNA phase separation associated with p‐bodies [43]. Based on these findings, we speculate that DDX6 can influence the depolymerization of Aβ aggregates through phase separation.

Although recent studies have demonstrated the benefits of directly targeting Aβ oligomers with monoclonal antibodies (e.g., lecanemab) in early AD patients, non‐negligible side effects include brain hemorrhage (17.3%), edema or effusion (12.6%), and headaches (11.1%) [15]. Additionally, approximately 28% of AD patients exhibited brain shrinkage after 18 months of lecanemab treatment [16]. Thus, DDX6‐targeting treatments in the early stages of Aβ aggregation could prevent the formation of Aβ oligomers and attenuate their neurotoxicity, potentially avoiding the side effects associated with strategies that directly target Aβ oligomers. Our results provide new insights into the neurotoxicity of Aβ oligomers in AD and establish a strong basis for the therapeutic potential of DDX6 targeting, along with a valuable foundation for further studies of DDX6 function.

## 4 Materials and Methods

### 4.1 Patients and Clinical Evaluations

Human brain tissue from South Central University's human brain bank was used in this study. Table S5 provides the following information for each patient: sample code, sex, age, Braak stage, Aβ phase, and postmortem interval.

### 4.2 Co‐Immunoprecipitation Assay

A Co‐IP kit (Thermo Fisher Scientific; #26149, USA) was used for the assay. Antibodies (2 µg) were crosslinked to a reinforced AminoLink coupling resin (20 µL of beads) and immobilized on a Pierce spin column at room temperature. Protein extracts (1 mg) were added to the spin column and incubated overnight at 4°C with rotation. The spin column was then transferred to a new collection tube, and 60 µL of elution buffer (pH 2.8, containing tertiary amine) was added. The column was centrifuged, and the eluate was analyzed to detect the presence of the target protein. Table S6 lists the antibodies used in this assay.

### 4.3 Western Blotting and Dot Blotting Assay

Protein concentrations in the co‐IP extracts were measured using a bicinchoninic acid assay (#23225, Thermo Fisher Scientific, USA). For each sample, 20 µL of protein was loaded, with the following amounts of human‐derived Aβ oligomers (AD1–AD6) used: 1.8 µg, 2.0 µg, 1.9 µg, 1.6 µg, 2.0 µg, and 1.8 µg, respectively. Equivalent amounts of co‐IP extracts were loaded into the remaining lanes. A 5× loading buffer was added to the protein samples to achieve a final concentration of 1×, and the samples were incubated at 95°C for 5 min [44]. For dot blotting [45], protein samples (2 µL per dot) were applied to a nitrocellulose membrane and incubated at room temperature for 30 min. Table S6 lists the antibodies used in this assay.

### 4.4 LC–MS/MS Analysis Protocol

An aliquot of each peptide mixture was loaded onto a trap column (in‐house‐fabricated column packed with a reversed‐phase ReproSil‐Pur C18‐AQ resin, 150 µm × 15 cm, C18, 3 µm, 100 Å, Dr. Maisch GmbH, Germany) connected to an analytical column (EASY‐Spray column, 50 m × 75 µm ID, PepMap RSLC C18, 2 µm, 100 Å, Thermo Scientific, USA) using the autosampler of an EASY‐nLC 1200 HPLC system (Thermo Fisher Scientific). The peptides were eluted using a gradient (solvent A: 0.1% formic acid in water; solvent B: 80% acetonitrile/0.1% formic acid in water; Sigma, Germany) directly into a Q Exactive Hybrid Quadrupole‐Orbitrap mass spectrometer (Thermo Fisher Scientific). The gradient program was as follows: 4%–8% solvent B over 2 min, 8%–28% solvent B over 43 min, 28%–40% solvent B over 10 min, 40%–95% solvent B over 1 min, and 95% solvent B for 10 min. The following mass spectrometry parameters were used: spray voltage, 2.2 kV; capillary temperature, 270°C; MS resolution, 70,000 at 400 *m*/*z*; MS precursor *m*/*z* range, 300.0–1800.0 *m*/*z*; activation type, high‐energy collision dissociation; normalized collision energy, 28.0; activation time, 66.000 s; and data‐dependent MS/MS, top 20 most intense peptide ions selected based on the preview scan in the Orbitrap.

### 4.5 Bioinformatic Analysis of MS Data via MaxQuant and SAINTexpress

The raw MS files (Figure S5) were analyzed and searched against species‐specific protein databases using MaxQuant (v1.6.2.10). The following parameters were applied: maximum missed cleavages, 2; precursor ion mass tolerance, 20 ppm; and MS/MS tolerance, 20 ppm. Peptides and proteins were filtered to achieve a false discovery rate of < 1% using a target‐decoy database strategy, and only proteins with at least two unique peptides were reported. Data were analyzed using the Significance Analysis of INTeractome (SAINTexpress) algorithm available at https://reprint‐apms.org, which determined whether each protein exhibited an actual interaction with Aβ oligomers [31]. SAINT is a probabilistic method for scoring protein–protein interactions in AP‐MS experiments, enabling the identification of proteins most likely to interact with target proteins. Proteins with SAINT scores > 0.5 were considered potential Aβ oligomer interaction partners (Tables S7 and S8).

### 4.6 IHC Staining and Analysis

Sections were dewaxed, subjected to antigen retrieval, and blocked. They were then incubated with primary antibody (1:500) overnight at 4°C, followed by secondary antibody (1:200). Nuclei were stained with DAPI (Beyotime; C1002, Shanghai, China) for 10 min in the dark [46]. Table S6 lists the antibodies used in this assay.

### 4.7 Assessment of Cell Viability Using CCK‐8 and YO‐PRO‐1/PI Assays

For antibody blockade experiments, antibodies targeting the four proteins and control IgG antibodies were separately added to human‐derived Aβ oligomer co‐IP extracts (2 µg) at a total antibody concentration ≤ 2 µg. These mixtures were incubated at 4°C for 1 h to block the respective proteins. HT‐22 cells (4000 cells per well) were seeded in 96‐well plates and treated with the four experimental groups in culture medium (100 µL) for 24 h. CCK‐8 reagent (10 µL, #C6005; NCM Biotech, China) was used to measure cell viability by determining optical density at 450 nm. To assess cell death, 100 µL of YO‐PRO‐1/PI working solution were added to each well and incubated for 1 h. The results were visualized using an inverted microscope (TS2; Nikon, Japan).

### 4.8 Mouse Brain Stereotaxic Injection

C57BL/６ mice (9 weeks old, 25–30 g body weight) were divided into three groups for brain stereotaxic injection: control, human‐derived Aβ oligomers, and DDX6 blockade (0.5 µg each). For hippocampal injections, the coordinates relative to bregma were anteroposterior = −2.0 mm, mediolateral = +1.7 mm, and dorsoventral = −1.4 mm. Injections were performed at a rate of 0.2 µL/min, and the needle was kept in place for 5 min after the injection.

### 4.9 Golgi Staining and Dendritic Analysis

Brain sections were immersed in Golgi developer for 30 min. Images were acquired using a tissue section scanner (3DHISTECH Pannoramic MIDI, China) and analyzed with Pannoramic Scanner software. Sholl analysis was used to measure total dendritic length and neuronal complexity [47].

### 4.10 Assessment of Learning and Memory by Y‐Maze, Novel Object Recognition, and Morris Water Maze Tests

Y‐maze: Mice were placed at the end of any arm and allowed to freely explore for 8 min [48]. Novel object recognition: Mice were acclimated to the environment for 15 min on Days 1–3. On Day 4, two yellow cubic objects were placed 20 cm apart at the bottom of the box, and mouse behavior was recorded for 5 min. On Day 5, one of the cubic objects was replaced with a red cylindrical object, and behavior was recorded again. Morris water maze: Mice were placed along the walls at the center of quadrants A, B, and C, and their movements were recorded for 1 min on Days 1–5. If a mouse failed to find the platform within 1 min, it was manually guided to the platform and allowed to remain there for 20 s. On Day 6, the platform was removed, and mice were placed at the center of quadrant A (opposite quadrant D); their movements were recorded for 1 min. Mouse behavior was analyzed using Tracking Master 4.4.2 software.

### 4.11 Protein Synthesis

The Aβ42 peptide was obtained from Abcam (#ab120301, USA). Aβ42 was solubilized using 1 mM hexafluoroisopropanol (#105228; Sigma‐Aldrich, Germany) and evaporated overnight in a desiccator to form a dry film. The film was then dissolved in dimethyl sulfoxide (#D8418; Sigma‐Aldrich) to a concentration of 5 mM, mixed with phosphate‐buffered saline, and incubated for 72 h at 4°C. The GST‐DDX6 protein was synthesized by KMD Bioscience Biotech Co., Ltd. (Tianjin, China). The biotin‐labeled short peptide was synthesized by Hubei Qiangyao Biotechnology Co., Ltd. (Hubei, China).

### 4.12 GST Pulldown

In accordance with the protocol provided in the GST pulldown kit (#21516; Thermo Fisher Scientific), 150 µg of GST‐DDX6 protein were rotated with 50 µL of glutathione agarose resin at 4°C for 3 h. The resin was then incubated overnight with 300 µg of recombinant Aβ oligomers or a negative control protein, washed with pH 8.0 glutathione elution buffer, and validated by dot blotting.

### 4.13 SPR Analysis

Nitrilotriacetic acid chips were prepared according to the standard operating procedures for OpenSPR instruments (Nicoya, Canada). Each chip was activated for 240 s with a mixture of 400 mM 1‐(3‐dimethylaminopropyl)‐3‐ethylcarbodiimide hydrochloride (EDC, #E1769‐25G; Sigma‐Aldrich) and 100 mM N‐hydroxysuccinimide (#130672‐25G; Sigma‐Aldrich) at a flow rate of 20 µL/min. DDX6 protein was diluted to 60 µg/mL in 10 mM sodium acetate and injected into the sample channel at a flow rate of 20 µL/min. The chip was deactivated for 240 s using 1 µM ethanolamine hydrochloride (#E6133‐100G; Sigma‐Aldrich). Aβ oligomers were diluted to six concentrations (10, 5, 2.5, 1.25, 0.625, and 0 µM) in assay buffer (1× phosphate‐buffered saline + 2% dimethyl sulfoxide). The oligomers were injected into the channel at a flow rate of 20 µL/min, allowed to bind for 240 s, and then dissociated for 360 s. Binding and dissociation steps were performed in assay buffer. Six analyte cycles were repeated in decreasing concentration order. Dynamic binding parameters and affinity constants were calculated using Biacore T200 evaluation software (GE, Sweden).

### 4.14 RT‐QuIC

ThT [49, 50] (#ab120751; Abcam, USA) was diluted in phosphate‐buffered saline (pH 7.4) to a final concentration of 20 µM. A small amount of 1% NH_4_OH (#105432; Merck, USA) was directly added to lyophilized solid Aβ42 (20 µM); the resulting Aβ42 solution was diluted to 1 mg/mL in phosphate‐buffered saline. Fibrillization of Aβ42 (20 µM) was performed in 0.1 M Tris–HCl buffer (pH 8.0) containing 20 µM ThT. Aβ42 oligomers (5 µM) were added to induce fibrillization. The reaction mixture was incubated at 37°C with intermittent oscillation (30 s every 30 min) for the specified duration. DDX6 (10 µM) was also added to the reaction system.

### 4.15 Peptide Array Technology

Activated polyethylene glycol‐modified cellulose membranes were placed on an AutoSpot peptide synthesizer (VERSA 110; Aurora Group Company, Canada) and programmed to automatically transfer Fmoc‐amino acid solutions (Chengdu Chengnuo Biotechnology Co., Ltd., China) to specific locations on the activated membranes. When synthesis was completed, side‐chain protecting groups were removed using a pethidine deprotection solution. In this experiment, each peptide array contained 13 columns and 13 rows (157 points arranged from left to right and top to bottom; the last row contained a single peptide point). After activation, the array membrane was incubated with blocking solution for 4 h. The recombinant Aβ oligomers (2.0 µg/mL) were diluted in blocking solution and incubated with peptide chips overnight at 4°C. The peptide chips were then incubated with primary antibody (1.0 µg/mL) for 2 h at room temperature with oscillation, followed by incubation with horseradish peroxidase‐labeled sheep anti‐rabbit secondary antibody for 2 h at room temperature. Finally, the peptide array chip was incubated with Pierce ECL chemiluminescence reagent (#32109; Thermo Fisher Scientific), and images were captured using the FX‐7 Digital Imaging Analyzer (VILBER FUSION FX7 Spectra; Vilber, France).

### 4.16 Statistical Analysis

Data were analyzed using GraphPad Prism 8.0 software (GraphPad Software, USA). Differences with *p*‐values < 0.05 were considered statistically significant. Protein band images were analyzed using ImageJ software (National Institutes of Health, USA). CaseViewer and ImageJ softwares were used for immunofluorescence image analysis.

## Author Contributions

Baian Chen, Jing Zhang, and Xiaoxin Yan designed and supervised the study. Xiaoxu Wang and Lu Dai had full access to all data and contributed to data acquisition, analysis, and interpretation. Xiaoxu Wang and Baian Chen wrote and revised the manuscript. Brain tissues were collected and analyzed by Qilei Zhang and Xiaoxin Yan. Administrative, technical, and material support were provided by Baian Chen, Jing Lu, Na Wu, Donghui Wu, Xinyuan Wang, Xia Meng, Qilei Zhang, and Xiaoxin Yan. All the authors critically reviewed the article and approved the final draft.

## Ethics Statement

The use of human tissues in this study was approved by the Institutional Review Boards of the School of Basic Medical Science, Central South University (Changsha, China) and Capital Medical University (Beijing, China). The respective approval numbers are 2020KT‐37 and Z2023SY001. In all cases, the use of human samples for research was conducted after written consent had been obtained from the patient and/or their legal guardian. All samples and data were anonymized and handled in accordance with relevant Chinese guidelines to protect patient privacy. All animal experiments were conducted in accordance with the Regulations on the Administration of Experimental Animals (China) and were approved by the Ethics Committee for Laboratory Animals of Capital Medical University (approval number: 7B866163‐941B‐40C3‐B78E‐0F89389F787F).

## Conflicts of Interest

The authors declare no conflicts of interest.

## Supporting information



Supporting Information

Supporting Information

Supporting Information

Supporting Information

## Data Availability

All data generated or analyzed during this study are included in this published article and its Supporting Information.

## References

[1] G. M. Shankar , S. Li , T. H. Mehta , et al., “Amyloid‐β Protein Dimers Isolated Directly From Alzheimer's Brains Impair Synaptic Plasticity and Memory,” Nature Medicine 14, no. 8 (2008): 837–842. 10.1038/nm1782 PMC2772133 18568035

[2] H. Zempel , E. Thies , E. Mandelkow , and E. M. Mandelkow , “Abeta Oligomers Cause Localized Ca(2+) Elevation, Missorting of Endogenous Tau Into Dendrites, Tau Phosphorylation, and Destruction of Microtubules and Spines,” Journal of Neuroscience 30, no. 36 (2010): 11938–11950. 20826658 10.1523/JNEUROSCI.2357-10.2010 PMC6633549

[3] Y. Gong , L. Chang , K. L. Viola , et al., “Alzheimer's Disease‐Affected Brain: Presence of Oligomeric A Beta Ligands (ADDLs) Suggests a Molecular Basis for Reversible Memory Loss,” PNAS 100, no. 18 (2003): 10417–10422. 12925731 10.1073/pnas.1834302100 PMC193576

[4] U. Sengupta , A. N. Nilson , and R. Kayed , “The Role of Amyloid‐β Oligomers in Toxicity, Propagation, and Immunotherapy,” Ebiomedicine 6 (2016): 42–49. 27211547 10.1016/j.ebiom.2016.03.035 PMC4856795

[5] M. Tolar , J. Hey , A. Power , and S. Abushakra , “Neurotoxic Soluble Amyloid Oligomers Drive Alzheimer's Pathogenesis and Represent a Clinically Validated Target for Slowing Disease Progression,” International Journal of Molecular Sciences 22, no. 12 (2021): 6355. 34198582 10.3390/ijms22126355 PMC8231952

[6] B. Zott , M. M. Simon , W. Hong , et al., “A Vicious Cycle of Beta Amyloid‐Dependent Neuronal Hyperactivation,” Science 365, no. 6453 (2019): 559–565. 31395777 10.1126/science.aay0198 PMC6690382

[7] S. J. C. Lee , E. Nam , H. J. Lee , M. G. Savelieff , and M. H. Lim , “Towards an Understanding of Amyloid‐β Oligomers: Characterization, Toxicity Mechanisms, and Inhibitors,” Chemical Society Reviews 46, no. 2 (2017): 310–323. 27878186 10.1039/c6cs00731g

[8] Y. Muñoz , A. C. Paula‐Lima , and M. T. Núñez , “Reactive Oxygen Species Released From Astrocytes Treated With Amyloid Beta Oligomers Elicit Neuronal Calcium Signals That Decrease Phospho‐Ser727‐STAT3 Nuclear Content,” Free Radical Biology and Medicine 117 (2018): 132–144. 29309895 10.1016/j.freeradbiomed.2018.01.006

[9] W. Wang , T. Hou , L. Jia , et al., “Toxic Amyloid‐β Oligomers Induced Self‐Replication in Astrocytes Triggering Neuronal Injury,” Ebiomedicine 42 (2019): 174–187. 30926423 10.1016/j.ebiom.2019.03.049 PMC6491655

[10] M. Townsend , G. M. Shankar , T. Mehta , D. M. Walsh , and D. J. Selkoe , “Effects of Secreted Oligomers of Amyloid β‐Protein on Hippocampal Synaptic Plasticity: A Potent Role for Trimers,” The Journal of Physiology 572, no. 2 (2006): 477–492. 16469784 10.1113/jphysiol.2005.103754 PMC1779683

[11] M. Shi , F. Chu , F. Zhu , and J. Zhu , “Impact of Anti‐Amyloid‐β Monoclonal Antibodies on the Pathology and Clinical Profile of Alzheimer's Disease: A Focus on Aducanumab and Lecanemab,” Frontiers in Aging Neuroscience 14 (2022): 870517. 35493943 10.3389/fnagi.2022.870517 PMC9039457

[12] K. I. Avgerinos , L. Ferrucci , and D. Kapogiannis , “Effects of Monoclonal Antibodies Against Amyloid‐β on Clinical and Biomarker Outcomes and Adverse Event Risks: A Systematic Review and Meta‐Analysis of Phase III RCTs in Alzheimer's Disease,” Ageing Research Reviews 68 (2021): 101339. 33831607 10.1016/j.arr.2021.101339 PMC8161699

[13] D. Jeremic , J. D. Navarro‐López , and L. Jiménez‐Díaz , “Efficacy and Safety of Anti‐Amyloid‐β Monoclonal Antibodies in Current Alzheimer's Disease Phase III Clinical Trials: A Systematic Review and Interactive Web App‐Based Meta‐Analysis,” Ageing Research Reviews 90 (2023): 102012. 37423541 10.1016/j.arr.2023.102012

[14] S. Salloway , S. Chalkias , F. Barkhof , et al., “Amyloid‐Related Imaging Abnormalities in 2 Phase 3 Studies Evaluating Aducanumab in Patients With Early Alzheimer Disease,” JAMA Neurology 79, no. 1 (2022): 13. 34807243 10.1001/jamaneurol.2021.4161 PMC8609465

[15] C. H. van Dyck , C. J. Swanson , P. Aisen , et al., “Lecanemab in Early Alzheimer's Disease,” New England Journal of Medicine 388, no. 1 (2023): 9–21. 36449413 10.1056/NEJMoa2212948

[16] J. Couzin‐Frankel , “Promising Alzheimer's Therapies Shrink Brains,” Science 380, no. 6640 (2023): 19. 37023200 10.1126/science.adi1220

[17] M. Filippi , G. Cecchetti , E. G. Spinelli , et al., “Amyloid‐Related Imaging Abnormalities and β‐Amyloid–Targeting Antibodies,” JAMA Neurology 79, no. 3 (2022): 291. 35099507 10.1001/jamaneurol.2021.5205

[18] M. Roytman , F. Mashriqi , K. Al‐Tawil , et al., “Amyloid‐Related Imaging Abnormalities: An Update,” American Journal of Roentgenology 220, no. 4 (2023): 562–574. 36321981 10.2214/AJR.22.28461

[19] T. Kim , G. S. Vidal , M. Djurisic , et al., “Human LilrB2 Is a β‐Amyloid Receptor and Its Murine Homolog PirB Regulates Synaptic Plasticity in an Alzheimer's Model,” Science 341, no. 6152 (2013): 1399–1404. 24052308 10.1126/science.1242077 PMC3853120

[20] F. H. Beraldo , V. G. Ostapchenko , F. A. Caetano , et al., “Regulation of Amyloid β Oligomer Binding to Neurons and Neurotoxicity by the Prion Protein‐mGluR5 Complex,” Journal of Biological Chemistry 291, no. 42 (2016): 21945–21955. 27563063 10.1074/jbc.M116.738286 PMC5063978

[21] L. M. Smith and S. M. Strittmatter , “Binding Sites for Amyloid‐β Oligomers and Synaptic Toxicity,” Cold Spring Harbor Perspectives in Medicine 7, no. 5 (2017): a024075. 27940601 10.1101/cshperspect.a024075 PMC5411685

[22] J. W. Um , H. B. Nygaard , J. K. Heiss , et al., “Alzheimer Amyloid‐β Oligomer Bound to Postsynaptic Prion Protein Activates Fyn to Impair Neurons,” Nature Neuroscience 15, no. 9 (2012): 1227–1235. 22820466 10.1038/nn.3178 PMC3431439

[23] T. Kawarabayashi , M. Shoji , L. H. Younkin , et al., “Dimeric Amyloid β Protein Rapidly Accumulates in Lipid Rafts Followed by Apolipoprotein E and Phosphorylated Tau Accumulation in the Tg2576 Mouse Model of Alzheimer's Disease,” The Journal of Neuroscience 24, no. 15 (2004): 3801–3809. 15084661 10.1523/JNEUROSCI.5543-03.2004 PMC6729359

[24] P. Rodriguez‐Rodriguez , A. Sandebring‐Matton , P. Merino‐Serrais , et al., “Tau Hyperphosphorylation Induces Oligomeric Insulin Accumulation and Insulin Resistance in Neurons,” Brain 140, no. 12 (2017): 3269–3285. 29053786 10.1093/brain/awx256

[25] S. Ghosh , R. Ali , and S. Verma , “Aβ‐Oligomers: A Potential Therapeutic Target for Alzheimer's Disease,” International Journal of Biological Macromolecules 239 (2023): 124231. 36996958 10.1016/j.ijbiomac.2023.124231

[26] E. Hlavanda , E. Klement , E. Kokai , et al., “Phosphorylation Blocks the Activity of Tubulin Polymerization‐Promoting Protein (TPPP): Identification of Sites Targeted by Different Kinases,” Journal of Biological Chemistry 282, no. 40 (2007): 29531–29539. 17693641 10.1074/jbc.M703466200

[27] S. Frykman , Y. Teranishi , J. Hur , et al., “Identification of Two Novel Synaptic γ‐Secretase Associated Proteins That Affect Amyloid β‐Peptide Levels Without Altering Notch Processing,” Neurochemistry International 61, no. 1 (2012): 108–118. 22521230 10.1016/j.neuint.2012.03.016

[28] J. Olah , O. Vincze , D. Virok , et al., “Interactions of Pathological Hallmark Proteins: Tubulin Polymerization Promoting Protein/p25, Beta‐Amyloid, and Alpha‐Synuclein,” Journal of Biological Chemistry 286, no. 39 (2011): 34088–34100. 21832049 10.1074/jbc.M111.243907 PMC3190826

[29] L. K. Habib , M. T. C. Lee , and J. Yang , “Inhibitors of Catalase‐Amyloid Interactions Protect Cells From β‐Amyloid‐Induced Oxidative Stress and Toxicity,” Journal of Biological Chemistry 285, no. 50 (2010): 38933–38943. 20923778 10.1074/jbc.M110.132860 PMC2998107

[30] A. Mendsaikhan , I. Tooyama , J. Bellier , et al., “Characterization of Lysosomal Proteins Progranulin and Prosaposin and Their Interactions in Alzheimer's Disease and Aged Brains: Increased Levels Correlate With Neuropathology,” Acta Neuropathologica Communications 7, no. 1 (2019): 215. 31864418 10.1186/s40478-019-0862-8 PMC6925443

[31] H. Choi , B. Larsen , Z. Lin , et al., “SAINT: Probabilistic Scoring of Affinity Purification–Mass Spectrometry Data,” Nature Methods 8, no. 1 (2011): 70–73. 21131968 10.1038/nmeth.1541 PMC3064265

[32] A. Breitkreutz , H. Choi , J. R. Sharom , et al., “A Global Protein Kinase and Phosphatase Interaction Network in Yeast,” Science 328, no. 5981 (2010): 1043–1046. 20489023 10.1126/science.1176495 PMC3983991

[33] G. Teo , G. Liu , J. Zhang , et al., “SAINTexpress: Improvements and Additional Features in Significance Analysis of INTeractome Software,” Journal of Proteomics 100 (2014): 37–43. 24513533 10.1016/j.jprot.2013.10.023 PMC4102138

[34] L. H. Mujawar , A. Moers , W. Norde , and A. van Amerongen , “Rapid Mastitis Detection Assay on Porous Nitrocellulose Membrane Slides,” Analytical and Bioanalytical Chemistry 405, no. 23 (2013): 7469–7476. 23912825 10.1007/s00216-013-7192-7

[35] S. G. De‐Simone , P. Napoleão‐Pêgo , L. A. L. Teixeira‐Pinto , et al., “IgE and IgG Epitope Mapping by Microarray Peptide‐Immunoassay Reveals the Importance and Diversity of the Immune Response to the IgG3 Equine Immunoglobulin,” Toxicon 78 (2014): 83–93. 24334152 10.1016/j.toxicon.2013.12.001

[36] R. Simon , H. Brylka , H. Schwegler , et al., “A Dual Function of Bcl11b/Ctip2 in Hippocampal Neurogenesis,” Embo Journal 31, no. 13 (2012): 2922–2936. 22588081 10.1038/emboj.2012.142 PMC3395096

[37] F. Negoita , M. Vavakova , J. Säll , J. Laurencikiene , and O. Göransson , “JUP/Plakoglobin Is Regulated by Salt‐Inducible Kinase 2, and Is Required for Insulin‐Induced Signalling and Glucose Uptake in Adipocytes,” Cellular Signalling 76 (2020): 109786. 32966883 10.1016/j.cellsig.2020.109786

[38] H. Park , T. Yamanaka , Y. Toyama , et al., “Hornerin Deposits in Neuronal Intranuclear Inclusion Disease: Direct Identification of Proteins With Compositionally Biased Regions in Inclusions,” Acta Neuropathologica Communications 10, no. 1 (2022): 1–17. 35246273 10.1186/s40478-022-01333-8 PMC8895595

[39] R. Bish , N. Cuevas‐Polo , Z. Cheng , et al., “Comprehensive Protein Interactome Analysis of a Key RNA Helicase: Detection of Novel Stress Granule Proteins,” Biomolecules 5, no. 3 (2015): 1441–1466. 26184334 10.3390/biom5031441 PMC4598758

[40] K. Saito , E. Kondo , and M. Matsushita , “MicroRNA 130 Family Regulates the Hypoxia Response Signal Through the P‐Body Protein DDX6,” Nucleic Acids Research 39, no. 14 (2011): 6086–6099. 21486751 10.1093/nar/gkr194 PMC3152344

[41] T. C. T. Michaels , A. Šarić , S. Curk , et al., “Dynamics of Oligomer Populations Formed During the Aggregation of Alzheimer's Aβ42 Peptide,” Nature Chemistry 12, no. 5 (2020): 445–451. 10.1038/s41557-020-0452-1 PMC7616962 32284577

[42] M. Hondele , R. Sachdev , S. Heinrich , et al., “DEAD‐Box ATPases Are Global Regulators of Phase‐Separated Organelles,” Nature 573, no. 7772 (2019): 144–148. 31435012 10.1038/s41586-019-1502-y PMC7617057

[43] L. Molitor , M. Klostermann , S. Bacher , et al., “Depletion of the RNA‐Binding Protein PURA Triggers Changes in Posttranscriptional Gene Regulation and Loss of P‐Bodies,” Nucleic Acids Research 51, no. 3 (2023): 1297–1316. 36651277 10.1093/nar/gkac1237 PMC9943675

[44] W. Huang , Y. Yan , Y. Liu , et al., “Exosomes With Low miR‐34c‐3p Expression Promote Invasion and Migration of Non‐Small Cell Lung Cancer by Upregulating Integrin α2β1,” Signal Transduction and Targeted Therapy 5, no. 1 (2020): 39. 32317629 10.1038/s41392-020-0133-y PMC7174429

[45] X. Zheng , Q. Wang , Y. Zhou , et al., “N‐Acetyltransferase 10 Promotes Colon Cancer Progression by Inhibiting Ferroptosis Through N4‐Acetylation and Stabilization of Ferroptosis Suppressor Protein 1 (FSP1) mRNA,” Cancer Communications 42, no. 12 (2022): 1347–1366. 36209353 10.1002/cac2.12363 PMC9759759

[46] R. He , Z. Wang , M. Cui , et al., “HIF1A Alleviates Compression‐Induced Apoptosis of Nucleus Pulposus Derived Stem Cells via Upregulating Autophagy,” Autophagy 17, no. 11 (2021): 3338–3360. 33455530 10.1080/15548627.2021.1872227 PMC8632345

[47] T. Jiang , Y. Li , S. He , et al., “Reprogramming Astrocytic NDRG2/NF‐kappaB/C3 Signaling Restores the Diabetes‐Associated Cognitive Dysfunction,” Ebiomedicine 93 (2023): 104653. 37329577 10.1016/j.ebiom.2023.104653 PMC10300300

[48] J. L. Verpeut , S. Bergeler , M. Kislin , et al., “Cerebellar Contributions to a Brainwide Network for Flexible Behavior in Mice,” Communications Biology 6, no. 1 (2023): 605. 37277453 10.1038/s42003-023-04920-0 PMC10241932

[49] L. Huang , T. Agrawal , G. Zhu , et al., “DAXX Represents a New Type of Protein‐Folding Enabler,” Nature 597, no. 7874 (2021): 132–137. 34408321 10.1038/s41586-021-03824-5 PMC8485697

[50] D. Yang , J. Li , Z. Li , et al., “Cardiolipin Externalization Mediates Prion Protein (PrP) Peptide 106–126‐Associated Mitophagy and Mitochondrial Dysfunction,” Frontiers in Molecular Neuroscience 16 (2023): 1163981. 37333615 10.3389/fnmol.2023.1163981 PMC10272765

