# Supplementary material for: DEAD‐Box Helicase 6 Blockade in Brain‐Derived Aβ Oligomers From Alzheimer's Disease Patients Attenuates Neurotoxicity
Source: MedComm (2020). 2025 Apr 24;6(5):e70156. doi: 10.1002/mco2.70156 (PMC12018770; doi:10.1002/mco2.70156)
Supplement: Supplementary file 1 — Supporting Information [file MCO2-6-e70156-s003.docx]

**DEAD-box RNA helicase DDX6 blockade in brain-derived Aβ oligomers from Alzheimer's disease patients** **attenuates neurotoxicity**

Xiaoxu Wang^1^#, Lu Dai^1^#, Na Wu^2^, Donghui Wu^1^, Xinyuan Wang^1^, Xia Meng^2^, Qilei Zhang^3^, Jing Lu^1，2^, Xiaoxin Yan^3^*, Jing Zhang^1，2^*, Baian Chen^1，2，4^*

^1^Department of Laboratory Animal Sciences; Capital Medical University, Beijing, 100069, China

^2^Laboratory Animal Resource Center, Capital Medical University, Beijing, 100069, China

^3^Department of Anatomy and Neurobiology, Central South University Xiangya School of Medicine, Changsha, Hunan 410013, China

^4^Center of Alzheimer's Disease, Beijing Institute of Brain Disorders, Capital Medical University, Beijing 100069, PR China

*Correspondence

Xiaoxin Yan: [yanxiaoxin@csu.edu.cn](mailto:yanxiaoxin@csu.edu.cn); Jing Zhang: [zhangjing2016@ccmu.edu.cn](mailto:zhangjing2016@ccmu.edu.cn); and Baian Chen: [baianchen@ccmu.edu.cn](mailto:baianchen@ccmu.edu.cn)

# Xiaoxu Wang and Lu Dai contributed equally to this work.


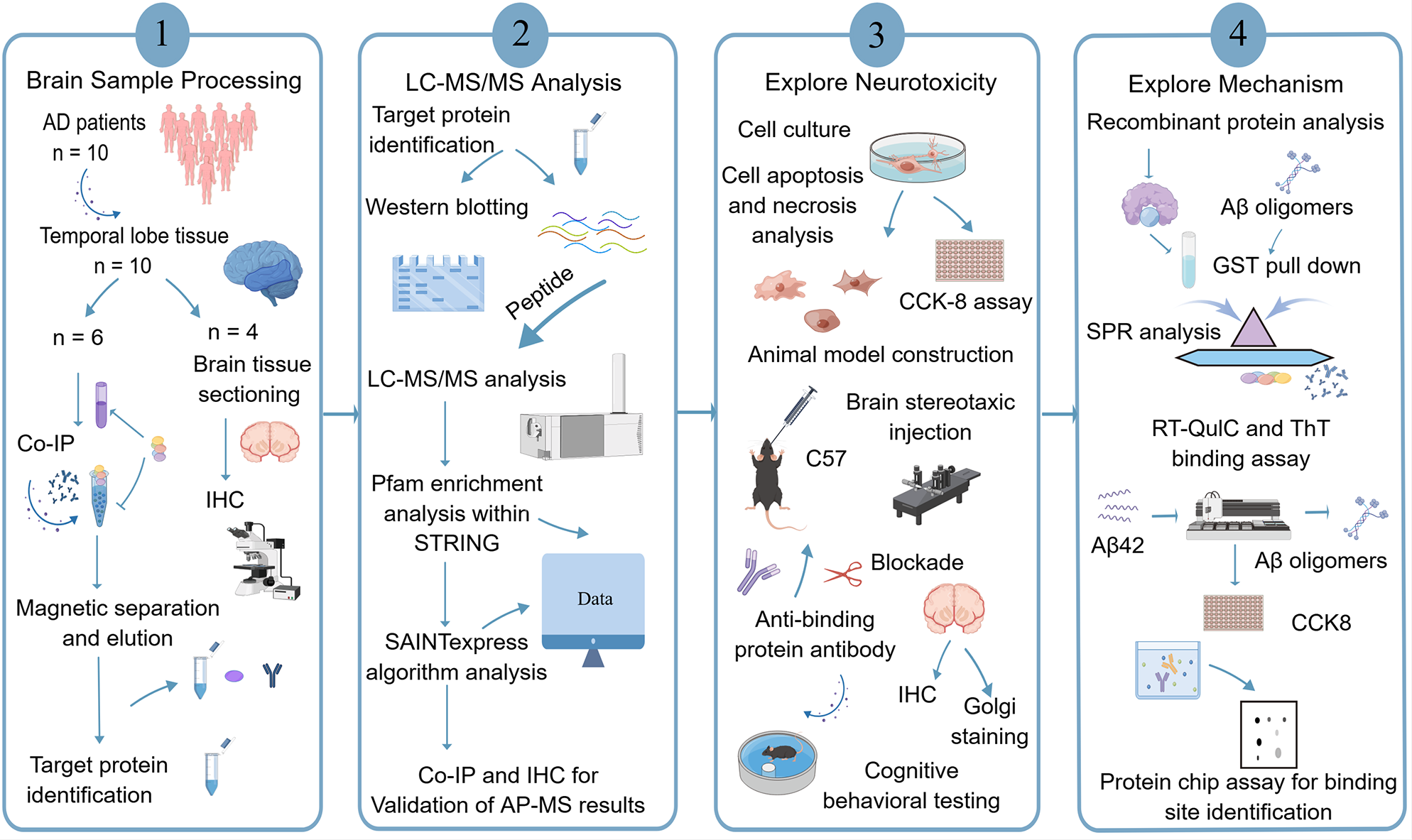


Figure S1. Exploratory approach and technical roadmap of this study.

1. Enrichment and extraction of Aβ oligomers: Co-immunoprecipitation (co-IP) technology was used to enrich and extract Aβ oligomers from total proteins isolated from the temporal lobe brain tissue of six Alzheimer's disease (AD) patients.

2. Protein identification and validation: First, liquid chromatography-tandem mass spectrometry (LC-MS/MS) was performed to identify all proteins present in AD brain-derived Aβ oligomers. Rigorous data analyses were then conducted to identify proteins most likely to interact with Aβ oligomers. These interactions were subsequently validated using co-IP and immunohistochemistry (IHC) staining on paraffin-embedded brain tissue slices from AD patients.

3. Identification of key toxic molecules: Binding proteins that affect the neurotoxicity of AD brain-derived Aβ oligomers were identified through both in vitro cell experiments (CCK-8 cytotoxicity assay, apoptosis and necrosis staining) and in vivo animal experiments (anti-binding protein antibody administration and cognitive behavioral testing).

4. Mechanistic investigation of key molecules: Multiple in vitro experiments were conducted to elucidate the mechanisms by which key molecules influence Aβ oligomer neurotoxicity. These experiments included GST-pull down, surface plasmon resonance (SPR), real-time quaking-induced conversion (RT-QuIC), and protein chip technology.


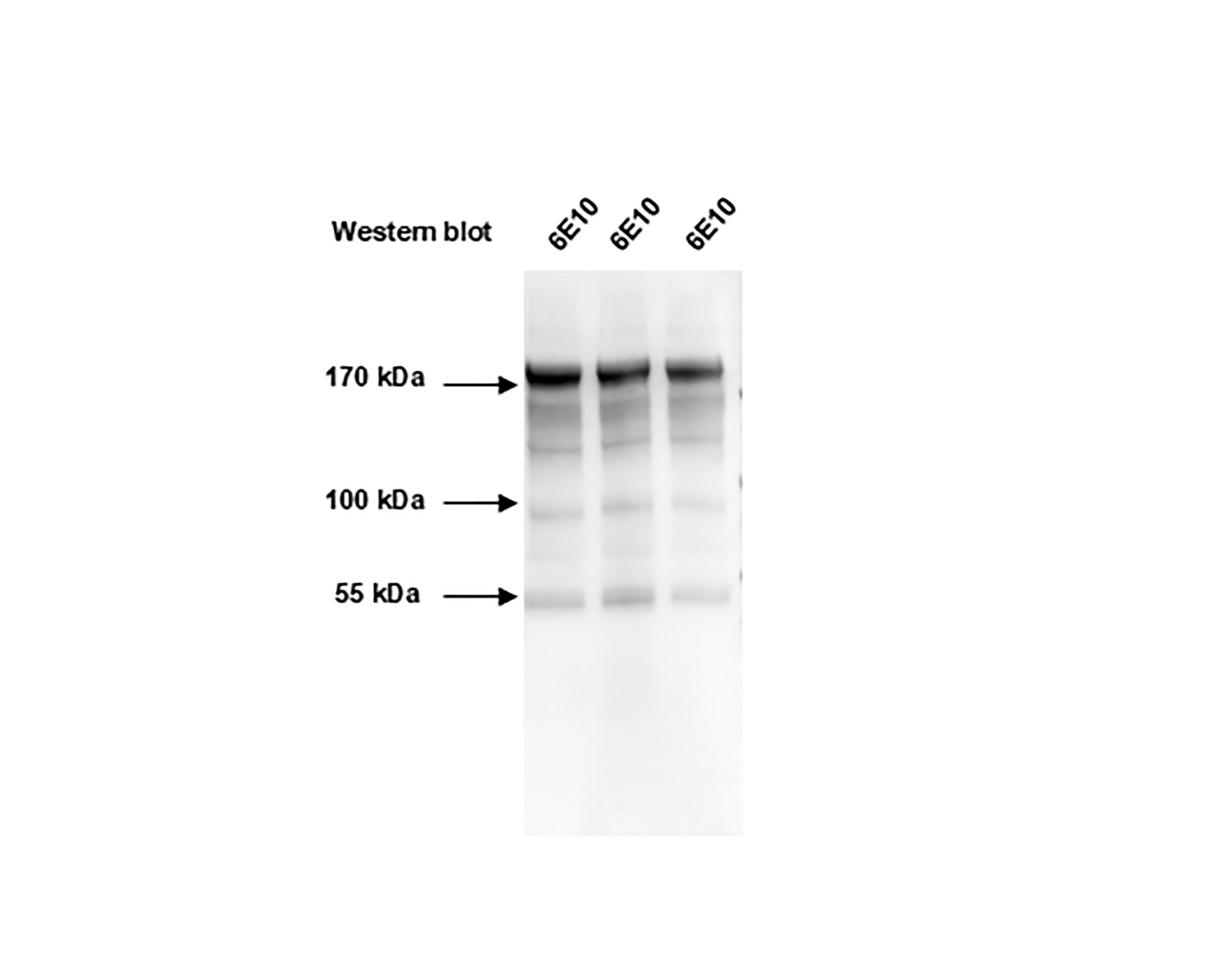


Figure S2. Western blotting analysis.

Western blotting was performed using the 6E10 antibody (anti-β-amyloid, 1-16, #39300; BioLegend, USA) to detect A11 antibody co-IP products extracted from the brain tissue of AD patients.


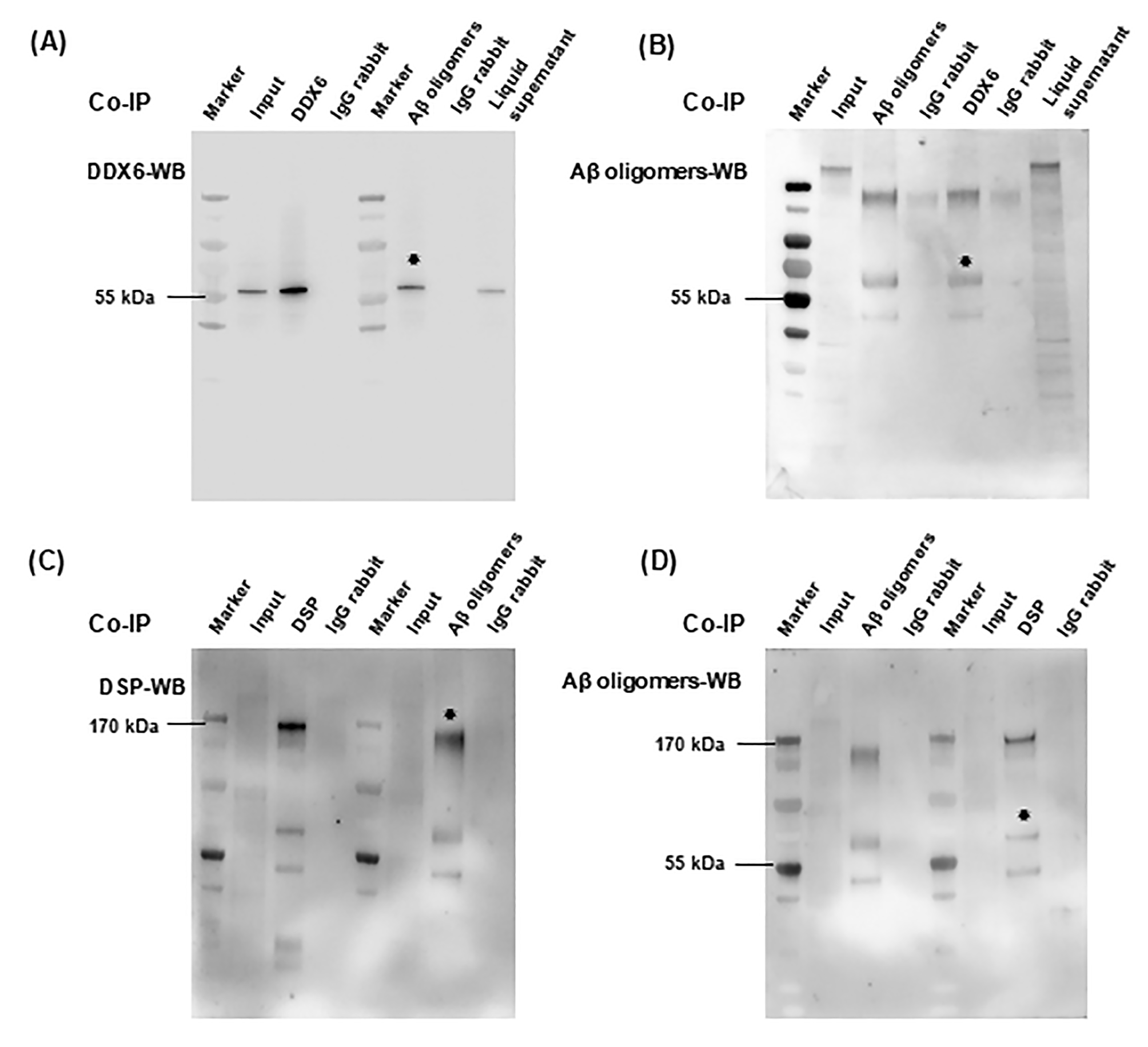
Figure S3. Co-IP and Western blotting analysis of the interaction between Aβ oligomers and DDX6/DSP in entire polyvinylidene fluoride membrane.

A. The polyvinylidene fluoride (PVDF) membrane was probed with the anti-DDX6 antibody, revealing enrichment of DDX6 (post-IP) with a molecular weight of 54 kDa. DDX6 was detected in the co-IP products of Aβ oligomers (indicated by “✸”). B. The PVDF membrane was probed with the Aβ oligomer antibody (A11), indicating enrichment of Aβ oligomers (post-IP) with bands observed at 55 kDa and 30 kDa. Aβ oligomers were detected in the co-IP products of DDX6 (indicated by “✸”). C. The PVDF membrane was probed with the anti-DSP antibody, showing enrichment of DSP (post-IP) with a molecular weight of 200 kDa. DSP was detected in the co-IP products of Aβ oligomers (indicated by “✸”). D. The PVDF membrane was probed with the anti-Aβ oligomer antibody, confirming the presence of Aβ oligomers in the co-IP products of DSP (indicated by “✸”).


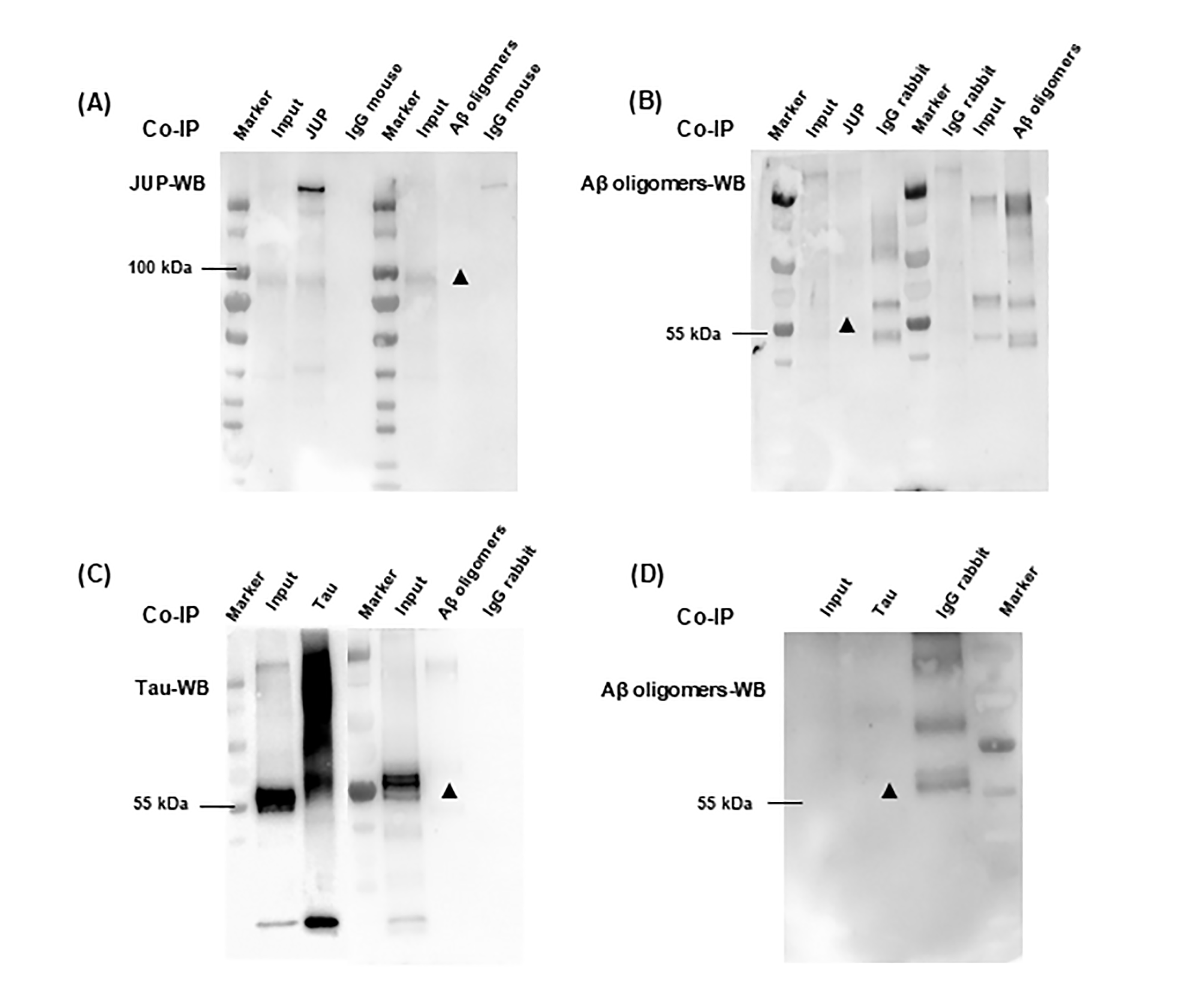


Figure S4. Co-IP and Western blotting analysis of the interaction between Aβ oligomers and JUP/Tau in entire polyvinylidene fluoride membrane.

A. The PVDF membrane was probed with the anti-JUP antibody, revealing no enrichment of JUP after IP. JUP, with a molecular weight of 80 kDa, was not detected in the co-IP products of Aβ oligomers (indicated by “▲”). B. The PVDF membrane was probed with the anti-Aβ oligomer antibody, indicating no detection of Aβ oligomers in the co-IP products of JUP (indicated by “▲”). C. The PVDF membrane was probed with the anti-tau antibody, demonstrating enrichment of tau protein after IP with a molecular weight of 55 kDa. However, tau was not detected in the co-IP products of Aβ oligomers (indicated by “▲”). D. The PVDF membrane was probed with the anti-Aβ oligomer antibody, showing no detection of Aβ oligomers in the co-IP products of tau protein (indicated by “▲”). Normal rabbit IgG or mouse IgG antibodies were used as controls to rule out non-specific binding.

AD1：
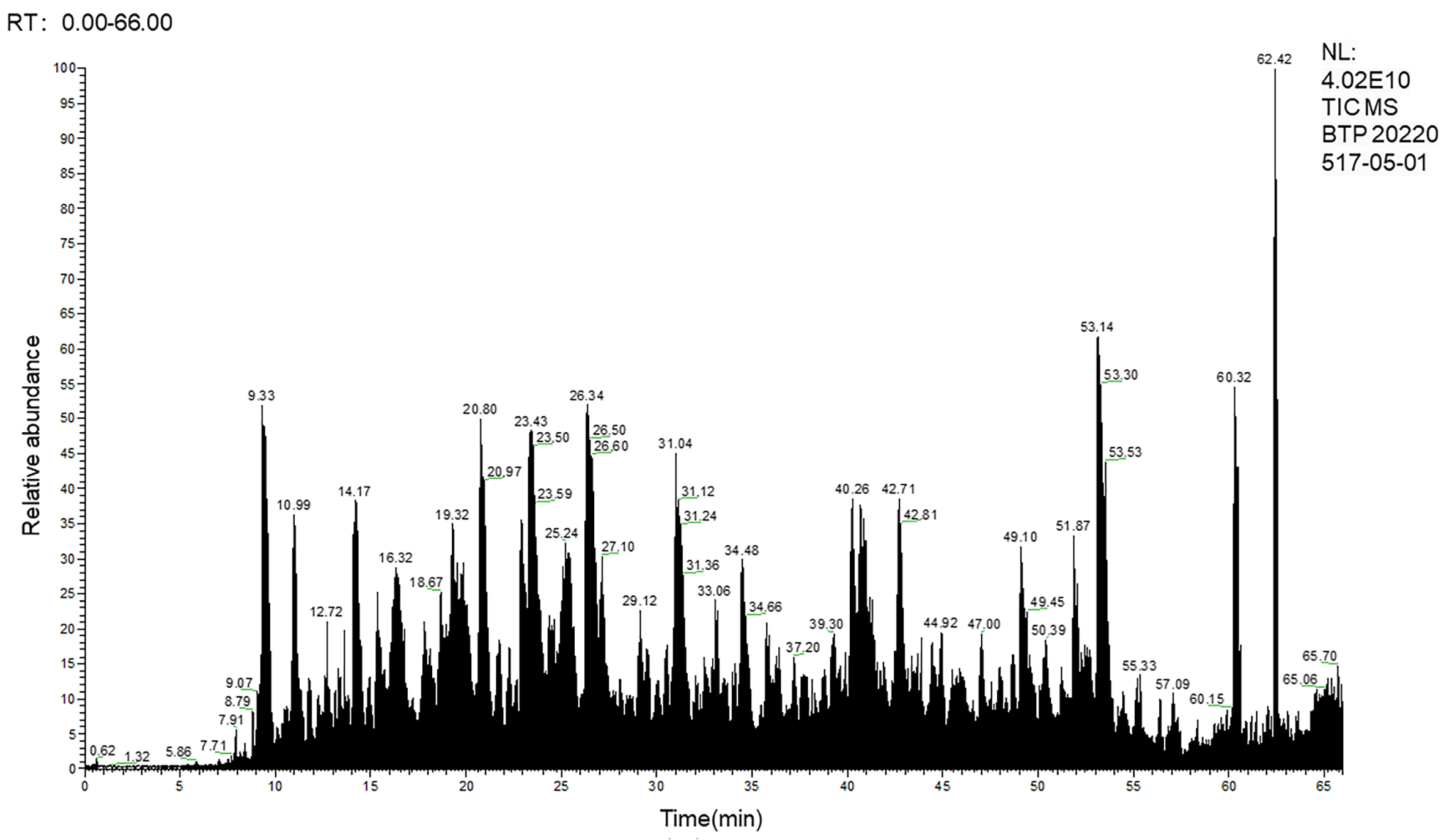


AD2：
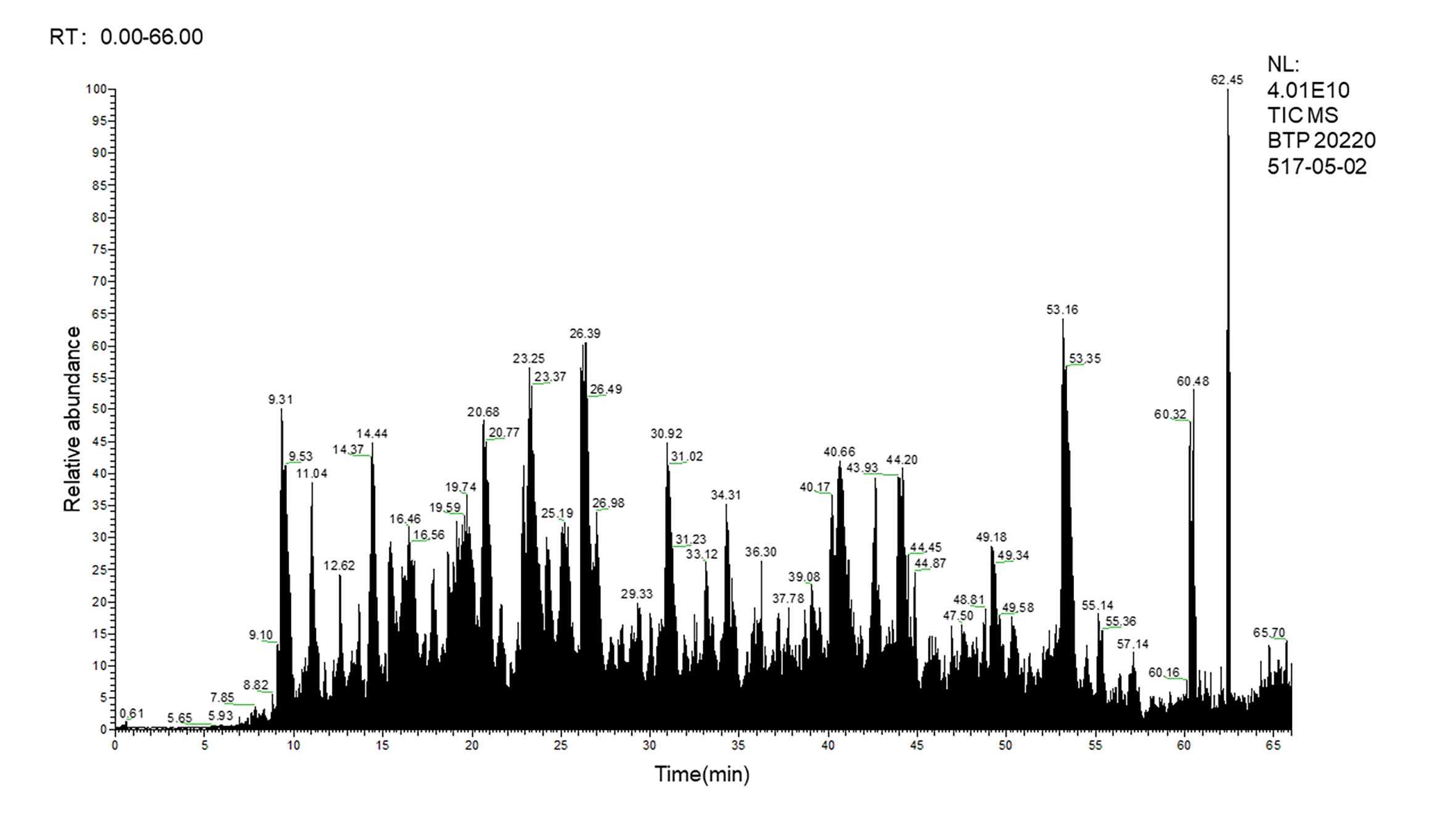


AD3：
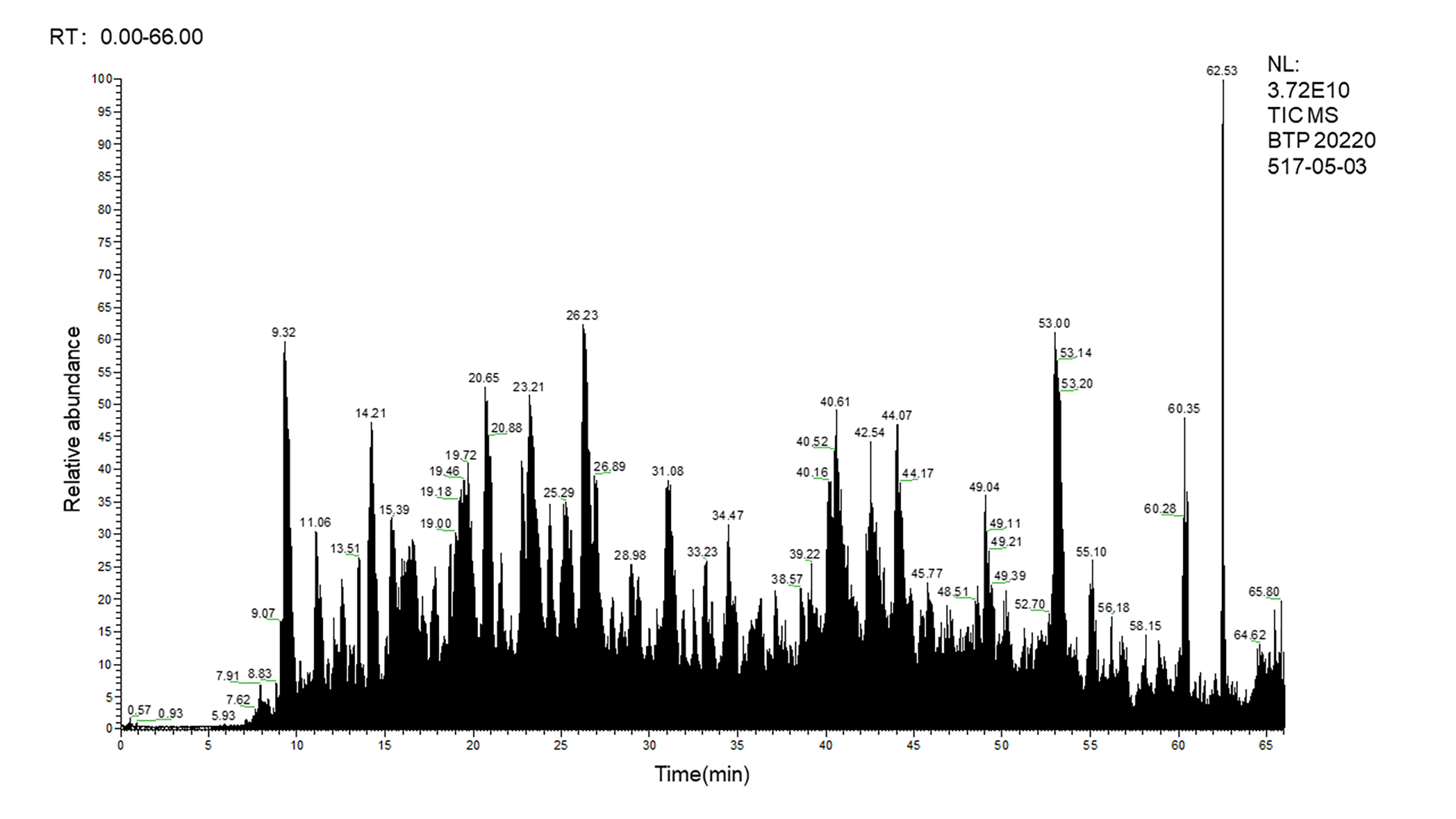


AD4：
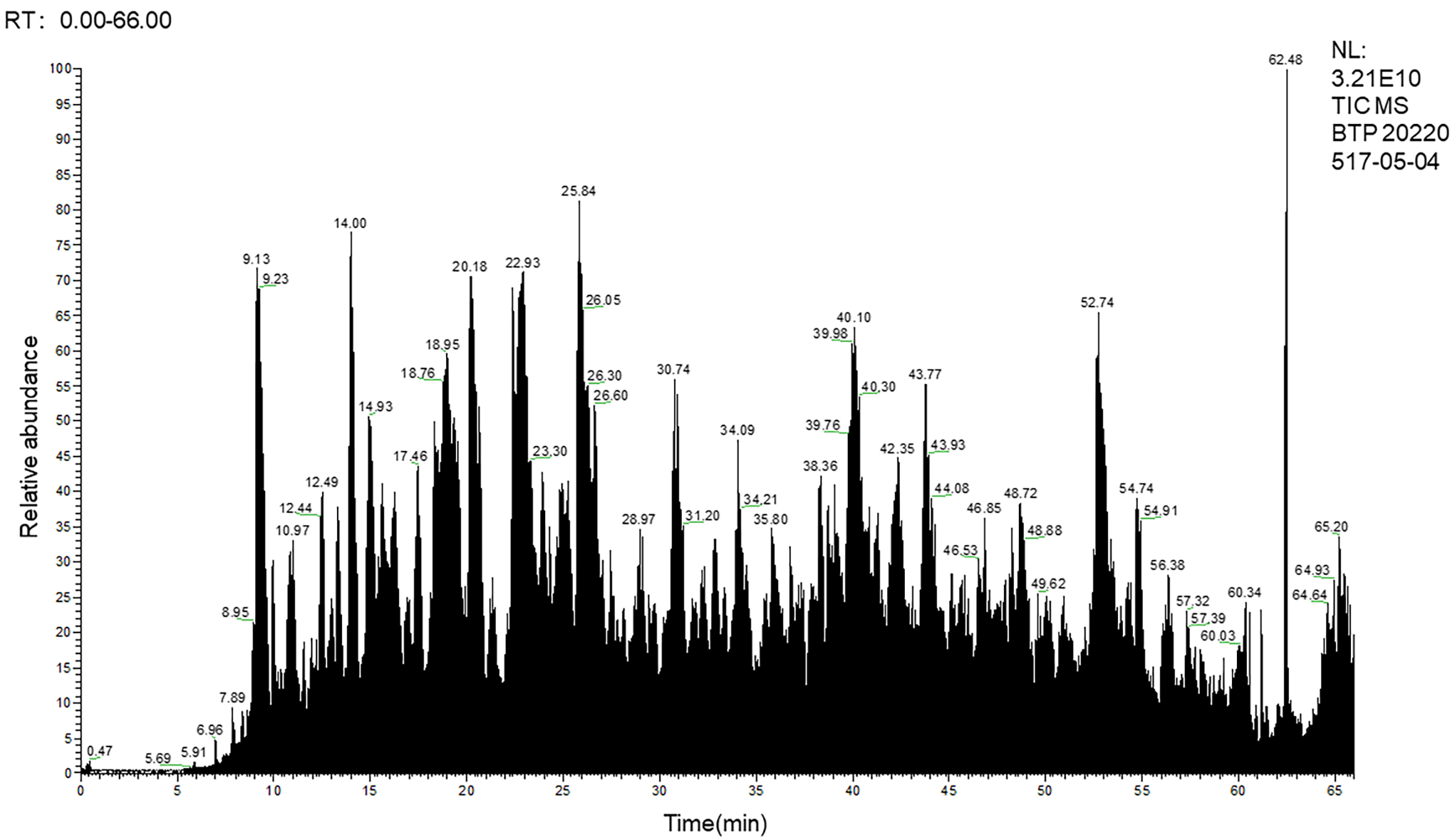


AD5：
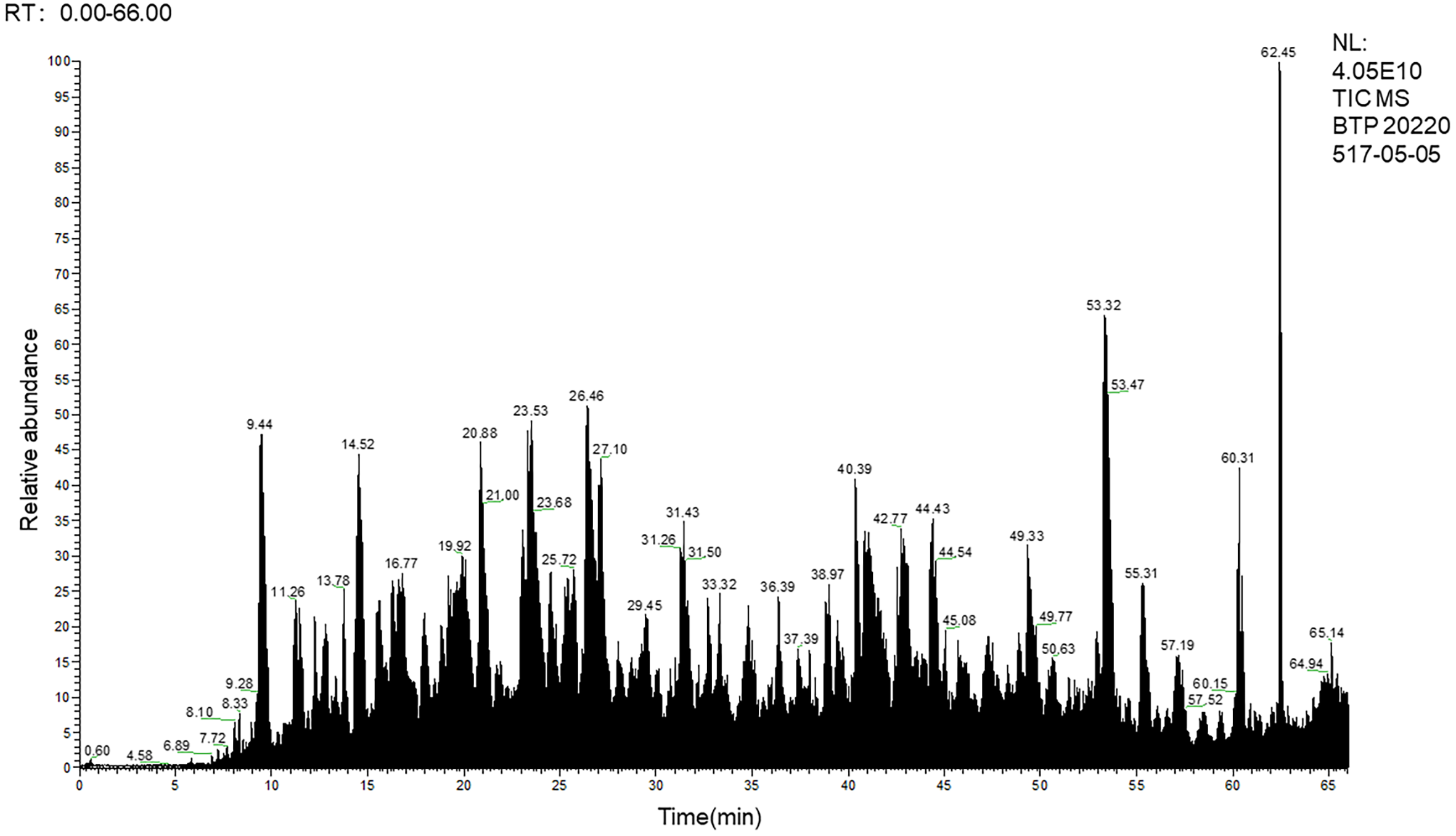


AD6：
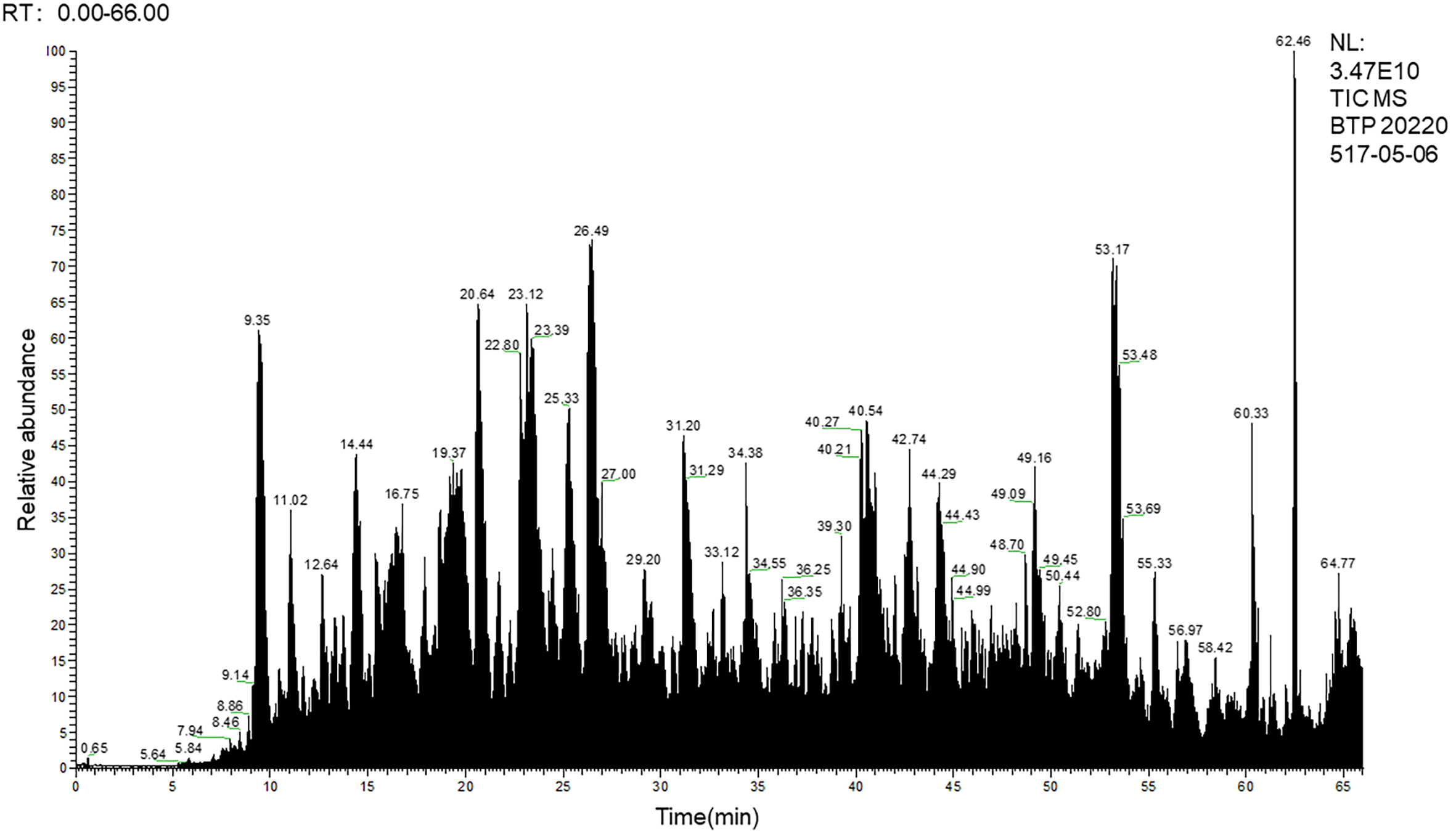


AD1-IgG control：
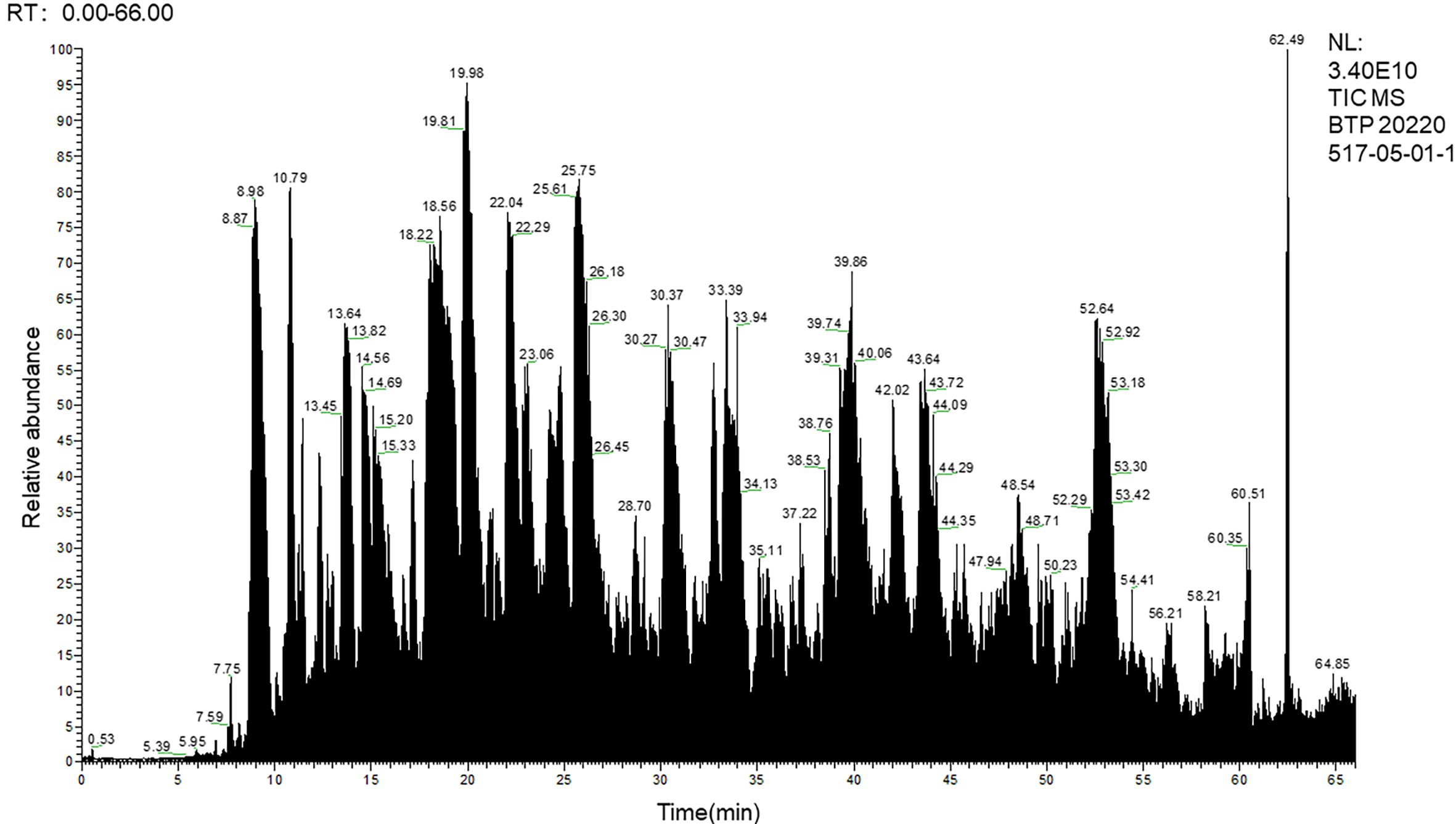


AD2-IgG control：
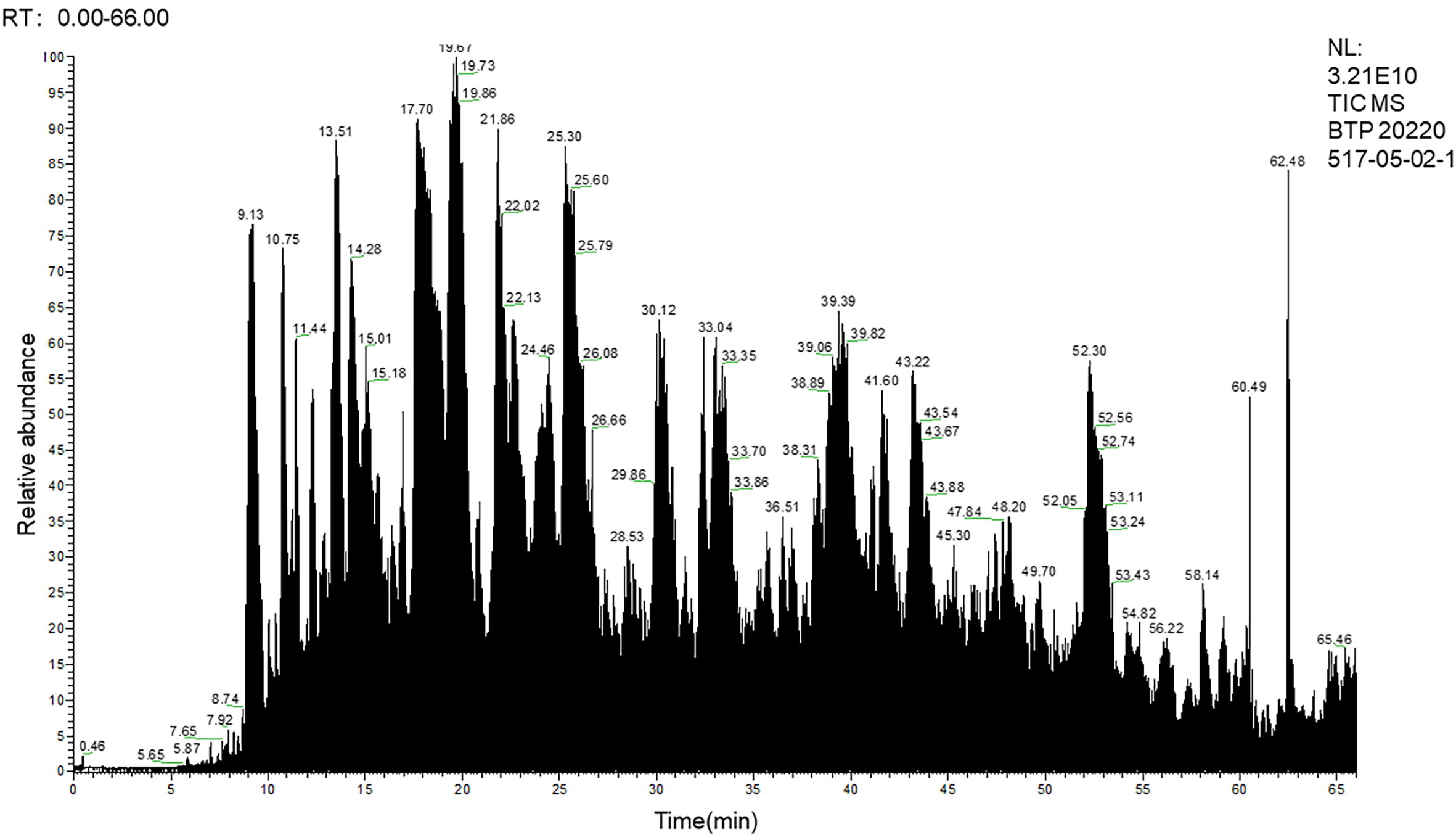


AD3-IgG control：
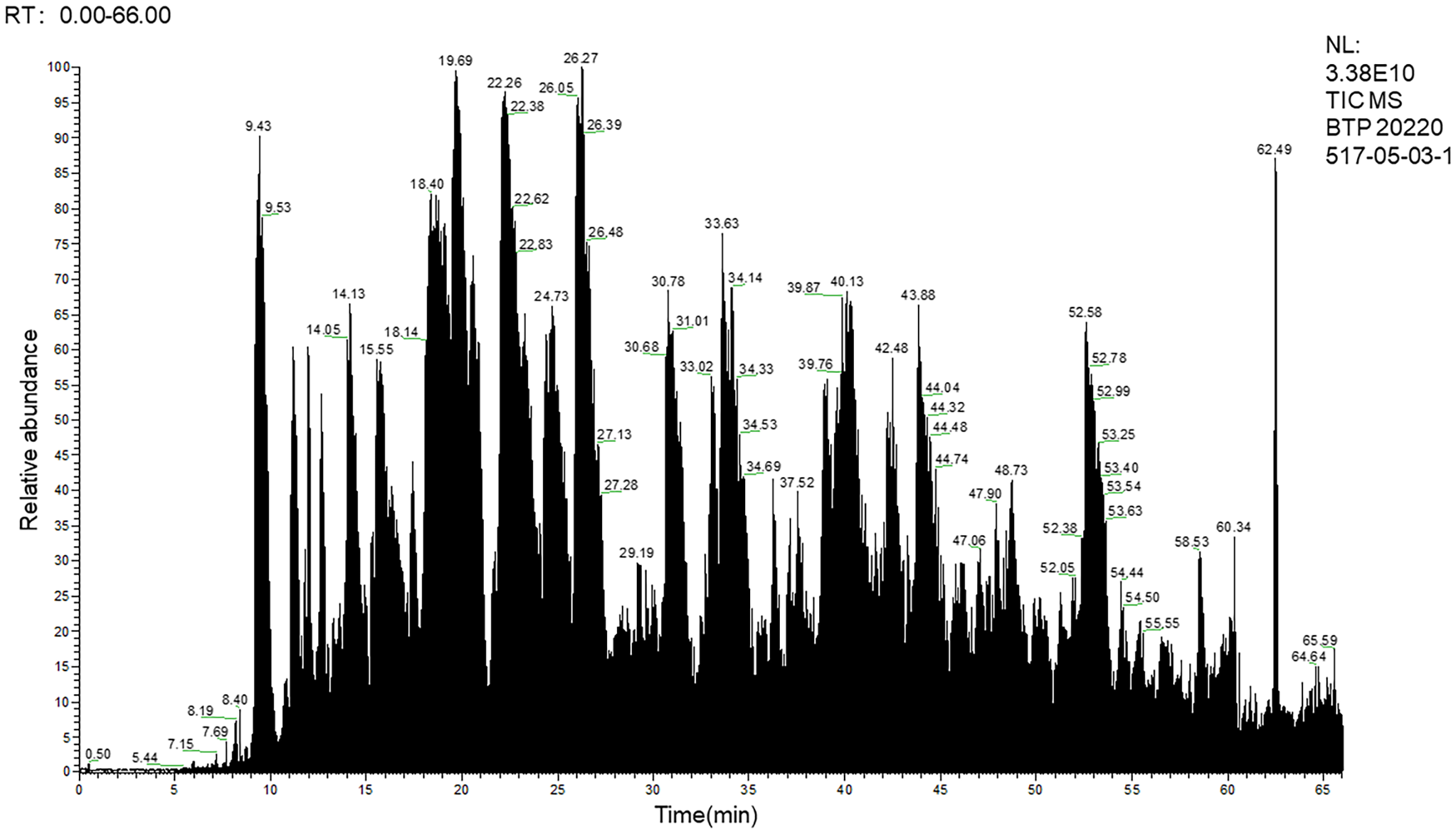


AD4-IgG control：
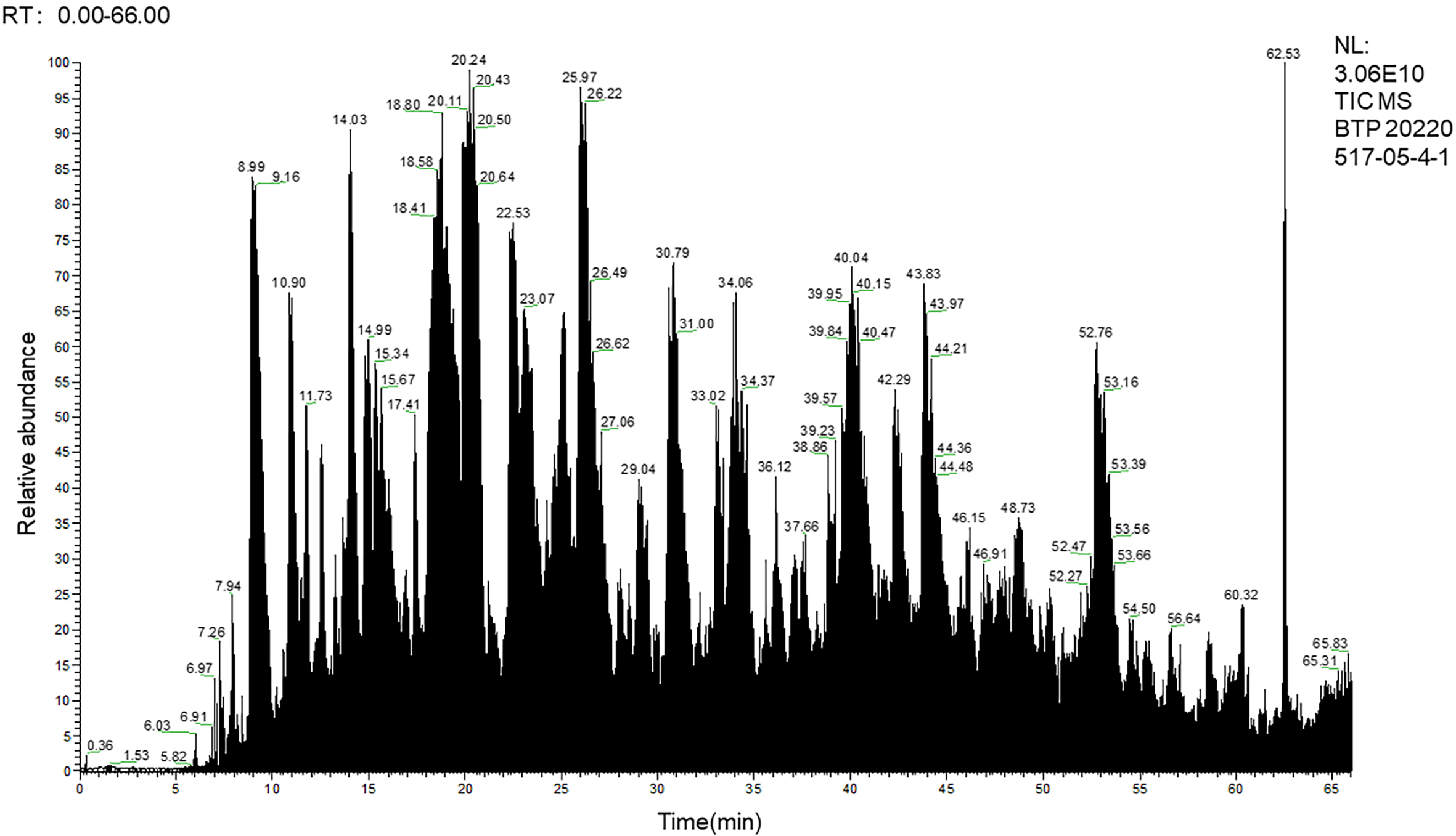


AD5-IgG control：
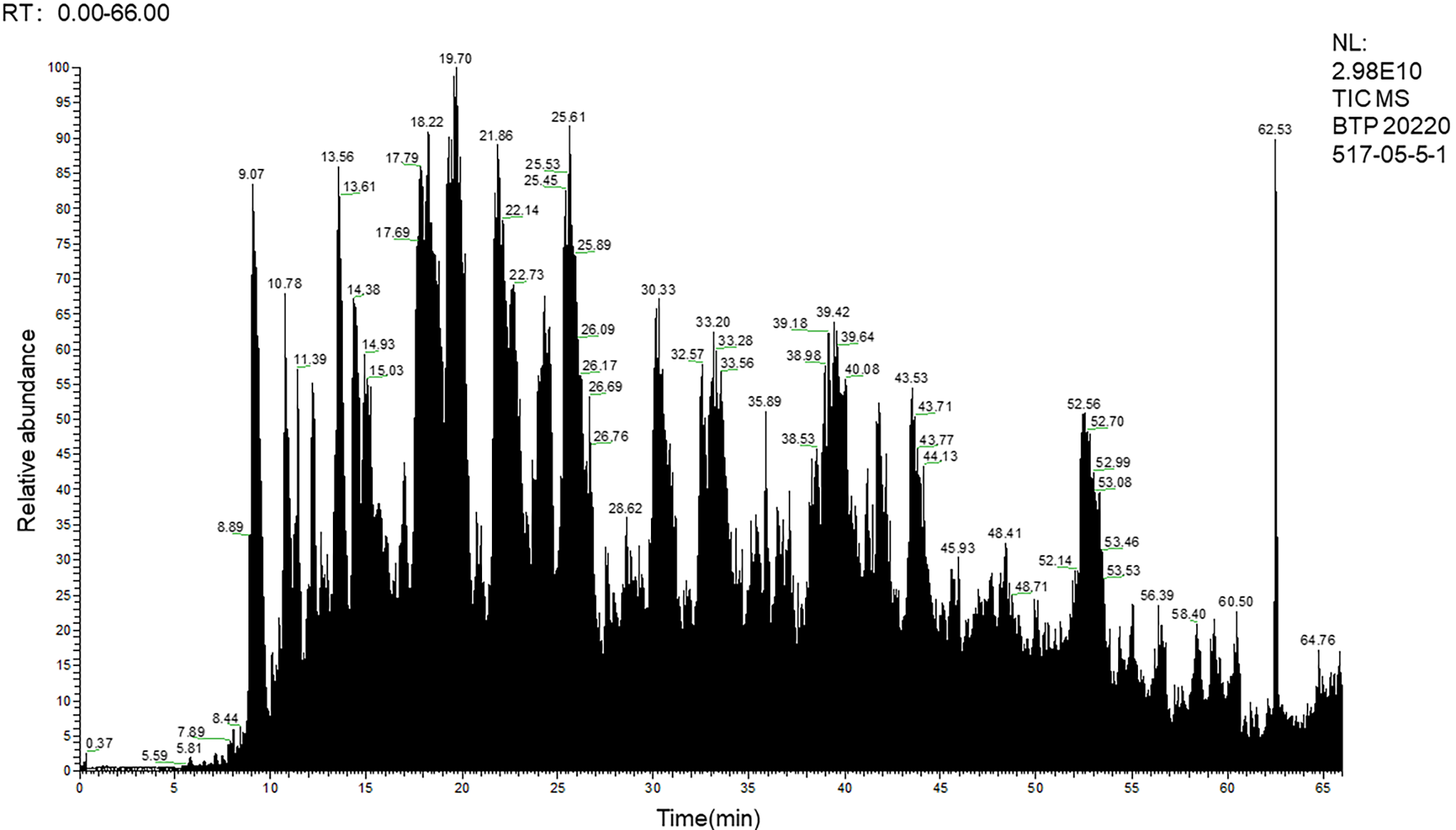


AD6-IgG control：
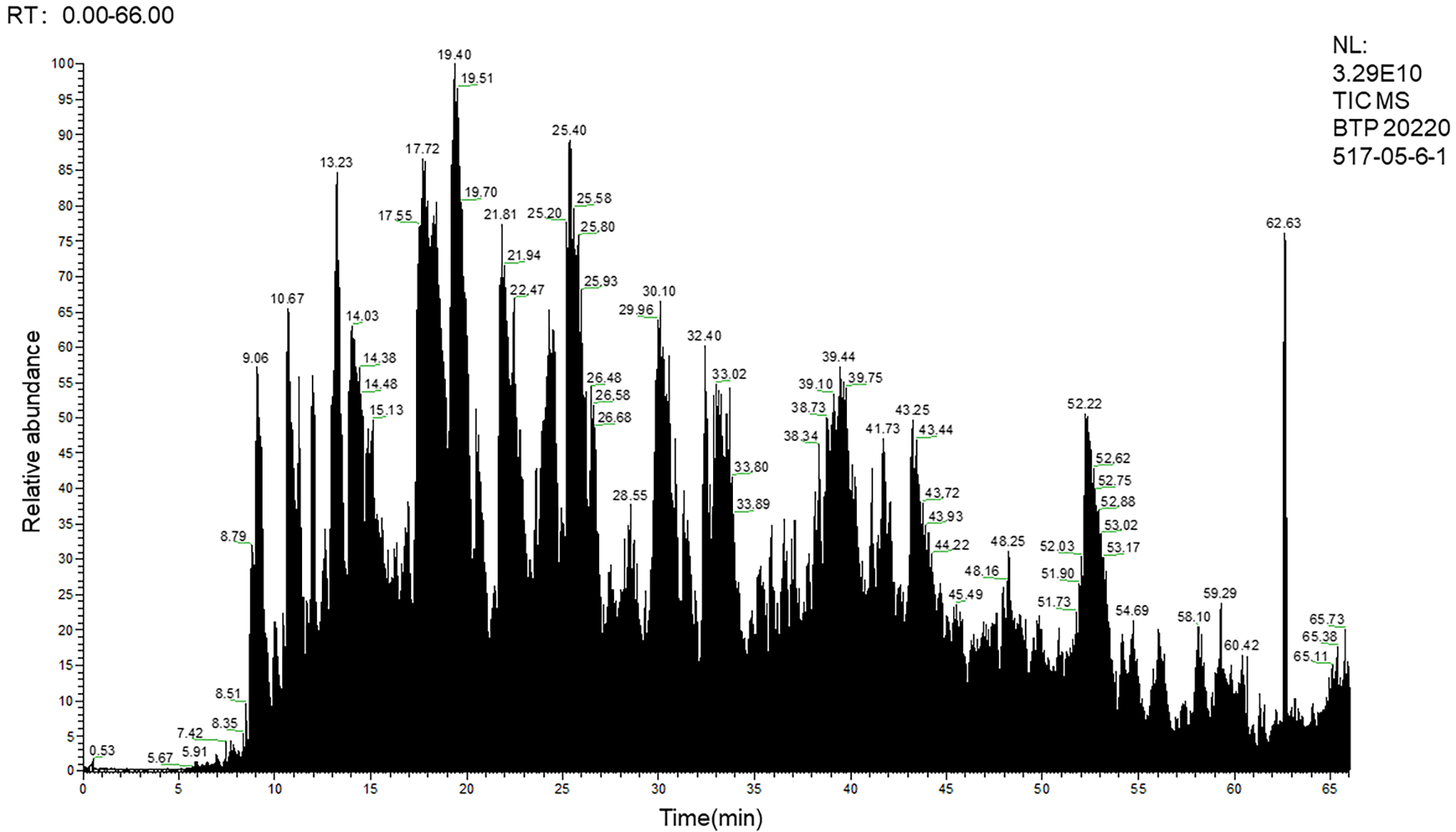


Figure S5. Mass spectrometry data acquisition. Raw data files were generated from mass spectrometry analysis and opened with Xcalibur software to view the total ion chromatograms for AD1–6 and their corresponding IgG control samples (AD1–6-IgG).

Table S1 Liquid chromatography-tandem masss spectrometry (LC-MS/MS) identified

all the proteins in human-derived Aβ oligomers and the control groups.

Table S2.

Two-factor repeated measures analysis of variance for the human-derived Aβ oligomers and control/DDX6 blockade groups. SD, standard deviation.

|  | Control (mean ± SD) | Human-derived Aβ oligomers (mean ± SD) | DDX6 blockade (mean ± SD) | Human derived Aβ oligomers and  Control | | | Human-derived Aβ oligomers and  DDX6 blockade | | |
| --- | --- | --- | --- | --- | --- | --- | --- | --- | --- |
|  |  |  |  | F | P | Partial η^2^ | F | P | Partial η^2^ |
| Day 1 | 47.09±8.23 | 45.72±14.55 | 44.61±13.46 |  |  |  |  |  |  |
| Day 2 | 40.36±13.09 | 46.28±10.84 | 34.03±12.15 |  |  |  |  |  |  |
| Day 3 | 32.06±9.37 | 40.89±16.22 | 35.58±17.50 |  |  |  |  |  |  |
| Day 4 | 34.06±15.90 | 40.58±17.44 | 28.86±18.35 |  |  |  |  |  |  |
| Day 5 | 26.00±15.13 | 39.89±8.46 | 28.11±11.55 |  |  |  |  |  |  |
| Groups |  |  |  | 5.129 | 0.034 | 0.196 | 5.763 | 0.025 | 0.208 |
| Days |  |  |  | 4.788 | 0.008 | 0.516 | 2.945 | 0.04 | 0.118 |
| Groups × days |  |  |  | 1.307 | 0.305 | 0.225 | 0.9 | 0.445 | 0.039 |

Table S3. Kinetic and affinity parameters.

| Parameter | Description | Result |
| --- | --- | --- |
| Ka (1/(M×s)) | Association rate constant | 3.42e3 |
| Kd (1/s) | Dissociation rate constant | 1.94e-2 |
| KD (M) | Equilibrium affinity constant | 5.67e-6 |

Table S4 Two-factor repeated measures analysis of variance for the DDX6+Aβ42+Aβ42 oligomers and Aβ42+Aβ42 oligomers

|  | DDX6+Aβ42+Aβ42 oligomers (mean ± SD) | Aβ42+Aβ42 oligomers (mean ± SD) | F | P | Partial η^2^ |
| --- | --- | --- | --- | --- | --- |
| Time 1 | 120440.00±2174.40 | 98354.00±3397.65 |  |  |  |
| Time 2 | 163205.33±17305.57 | 137802.00±10731.52 |  |  |  |
| Time 3 | 165687.33±17376.55 | 135795.33±9888.56 |  |  |  |
| Time 4 | 166625.67±17046.37 | 135025.33±10555.61 |  |  |  |
| Time 5 | 168205.00±16365.47 | 135671.33±10930.83 |  |  |  |
| Groups |  |  | 9.09 | 0.039 | 0.694 |
| Times |  |  | 55.8 | 0.001 | 0.933 |
| Groups × times |  |  | 0.789 | 0.428 | 0.165 |

Table S5. Patient characteristics.

| PatientID | Sex | tau  Braak stage  Age  (years) | | β-amyloid plaque Thal phase | Postmortem interval (h) |
| --- | --- | --- | --- | --- | --- |
| AD1 | Female | 88 | V | 3 | 9 |
| AD2 | Male | 82 | IV | 4 | 9.5 |
| AD3 | Male | 70 | IV | 3 | 8 |
| AD4 | Male | 83 | V | 4 | 12 |
| AD5 | Male | 89 | III | 1 | 8 |
| AD6 | Male | 86 | III | 4 | 6 |
| AD7 | Male | 85 | III | 2 | n/a |
| AD8 | Male | 91 | V | 3 | n/a |
| AD9 | Female | 95 | IV | 3 | n/a |
| AD10 | Female | 64 | I | 2 | n/a |

NOTE. n/a = not available

Table S6. Antibody information.

| Antibody | Product number | Brand |
| --- | --- | --- |
| A11(Aβ oligomers) | AHB0052 | Thermo Fisher Scientific |
| DDX6 | ab70455 | Abcam |
| DSP | ab109445 | Abcam |
| Tau | ab254256 | Abcam |
| JUP | ab305241 | Abcam |
| HRNR | ab78909 | Abcam |
| Isotype control anti-rabbit IgG antibody | ab172730 | Abcam |
| Isotype control anti-rabbit IgG antibody | 2729S | Cell Signaling Technology |
| Isotype control anti-mouse IgG antibody | ab18431 | Abcam |
| Horseradish peroxidase-conjugated secondary antibody (goat anti-mouse IgG) | ZB-2305 | ZSGI-BIO |
| Horseradish peroxidase-conjugated secondary antibody (goat anti-rabbit IgG) | ZB-2301 | ZSGI-BIO |
| NFH | ab8135 | Abcam |
| PSD95 | ab18258 | Abcam |
| Goat anti-rabbit IgG secondary antibody | ab6717 | Abcam |

Table S7 The fold change (FC value) of protein expression between the human-derived

Aβ oligomers and the control groups.

Table S8 The actual interaction values of proteins with human-derived Aβ oligomers were obtained through the Significance Analysis of INTeractome (SAINTexpress) algorithm.
